# Deregulation upon DNA damage revealed by joint analysis of context-specific perturbation data

**DOI:** 10.1186/1471-2105-12-249

**Published:** 2011-06-21

**Authors:** Ewa Szczurek, Florian Markowetz, Irit Gat-Viks, Przemysław Biecek, Jerzy Tiuryn, Martin Vingron

**Affiliations:** 1 Computational Molecular Biology Department, Max Planck Institute for Molecular Genetics, Ihnestrasse 73, 14195 Berlin,Germany; 2 International Max Planck Research School for Computational Biology and Scientific Computing, Berlin, Germany; 3 Faculty of Mathematics, Informatics and Mechanics, University of Warsaw, Banacha 2, 02-097 Warsaw, Poland; 4 Cancer Research UK Cambridge Research Institute, Cambridge, United Kingdom, 5Broad Institute of MIT and Harvard, 7 Cambridge Center, Cambridge, MA 02142, USA; 5 Broad Institute of MIT and Harvard, 7 Cambridge Center, Cambridge, MA 02142, USA

## Abstract

**Background:**

Deregulation between two different cell populations manifests itself in changing gene expression patterns and changing regulatory interactions. Accumulating knowledge about biological networks creates an opportunity to study these changes in their cellular context.

**Results:**

We analyze re-wiring of regulatory networks based on cell population-specific perturbation data and knowledge about signaling pathways and their target genes. We quantify deregulation by merging regulatory signal from the two cell populations into one score. This joint approach, called JODA, proves advantageous over separate analysis of the cell populations and analysis without incorporation of knowledge. JODA is implemented and freely available in a Bioconductor package 'joda'.

**Conclusions:**

Using JODA, we show wide-spread re-wiring of gene regulatory networks upon neocarzinostatin-induced DNA damage in Human cells. We recover 645 deregulated genes in thirteen functional clusters performing the rich program of response to damage. We find that the clusters contain many previously characterized neocarzinostatin target genes. We investigate connectivity between those genes, explaining their cooperation in performing the common functions. We review genes with the most extreme deregulation scores, reporting their involvement in response to DNA damage. Finally, we investigate the indirect impact of the ATM pathway on the deregulated genes, and build a hypothetical hierarchy of direct regulation. These results prove that JODA is a step forward to a systems level, mechanistic understanding of changes in gene regulation between different cell populations.

## Background

Molecular profiling of cells sampled from healthy patients and patients suffering from diseases led to the discovery of signatures of deregulated genes, i.e., distinctive expression patterns of genes that are differentially regulated and thus change expression between these two populations of cells. Such deregulated genes facilitate classification into different tumors [1-5], define new cancer subtypes and can serve as predictors of tumor differentiation stages and patient survival [6-10].

In recent years, the focus of research has moved from analyzing differentially expressed genes to analyzing differential regulatory networks [11]. These approaches are based on the observation that cellular adaptation to different environments and stimuli [12], to changes induced by diseases [13-16] or gene mutations [17], as well as to developmental processes [18] results in gains or losses of interactions in the molecular networks of the cell [19]. For example, Workman *et al*. [12] showed extensive re-wiring of gene regulatory networks in yeast cells undergoing DNA damage by using genome-wide measurements of gene expression upon transcription factor (TF) perturbations, as well as TF binding to DNA.

Computational and statistical analysis of changes in network structure between two cell populations has become a rapidly expanding field of research [11]. Many methods have been developed to infer differential interactions from gene expression data, either based on linear measures of correlation [20,14,22] and regression [23] or non-linear information theoretic criteria [13]. Additional to methods comparing two cellular populations, there are dynamic approaches to infer re-wiring over time [24,18].

However, these extant approaches to analyze deregulation between two different cell populations do mostly not take into account available knowledge about cellular signaling pathways nor their transcriptional targets, which may also differ between the cell populations. For example, Mani *et al*. [13] and Taylor *et al*. [14] take as input a static interactome, which is not specific for the two cell populations, to discover loss or gain of expression correlation between its nodes. The advanced approach of Workman *et al*. [12] could be further improved by incorporating prior information about the signaling pathways that are differentially activated upstream of the re-wired gene regulatory network, and the complementarity between the TF DNA-binding and the TF perturbation data.

Here, we present a novel approach to assess re-wiring in two cell populations that combines two key ideas: (1) we analyze the effects of pathway-targeted experimental single-gene perturbations and (2) we explicitly include knowledge of pathway topologies and their downstream targets. In this way, our approach facilitates research in a particular context of the biological system under study, implementing the concept of data analysis that gains power from incorporation of knowledge [25]. Our knowledge-based approach is designed for quantifying *deregulation*, i.e., changes in gene regulatory network between two different populations of cells. It performs joint analysis of perturbation data from the two cell populations, and is referred to as *joint deregulation analysis *(JODA) throughout the text. The cell populations may correspond to healthy and diseased cells, or diseased cells in two different stages, or, more generally, cells exposed to two different external stimuli, with different cellular signaling and downstream transcriptional targets.

JODA analyzes high-throughput perturbation experiments, where genome-wide expression is measured upon single-gene knock-outs or knock-downs. It is assumed that the set of perturbed genes is composed of regulators, i.e., genes active in signaling or gene regulation systems of the analyzed cells. The perturbations need to be performed on the same set of regulators in both cell populations.

The first kind of knowledge given as input to JODA is the information about the topology of the signaling pathways active in the two cell populations. The pathway topologies are graphs, which represent regulators with nodes and the known signaling relations between the regulators with edges. Internally, based on the given pathway topologies, JODA builds two binary matrix models (one per each cell population). The models are used by the algorithm to determine which perturbation experiments affect which regulators in the pathways. Second, JODA takes as input the known regulator-target gene relations downstream of the pathways. Those relations, when available, are given for those regulators, which are also TFs, and their known target genes. Since both the signaling and regulatory relations may differ between the two cell populations, they need to be provided for both of them separately.

The output of JODA are deregulation scores that quantify deregulation using the difference in perturbation effects in the two cell populations. An up-regulation effect indicates (possibly indirect) inhibition, and down-regulation indicates activation by the perturbed regulator. The most extreme deregulation scores are assigned to those genes which switch regulatory mechanism between the cell populations and show a different perturbation effect in the one cell population than in the other. We show that JODA performs better than investigating gene regulation in each cell population separately: with the deregulation scores, JODA prioritizes genes that are more enriched in those Gene Ontology (GO [26]) terms which are important for the switch between the compared cell populations. Similarly, functionally important genes can be missed when deregulation is analyzed without incorporation of prior knowledge, but based only on differences in expression correlation, adapting the ideas of Mani *et al*. [13] and Taylor *et al*. [14]. An R package 'joda', implementing the JODA algorithm, is released by Bioconductor [27]. A short summary of the package functionality and its demo are available at http://joda.molgen.mpg.de/.

**Figure 1:**
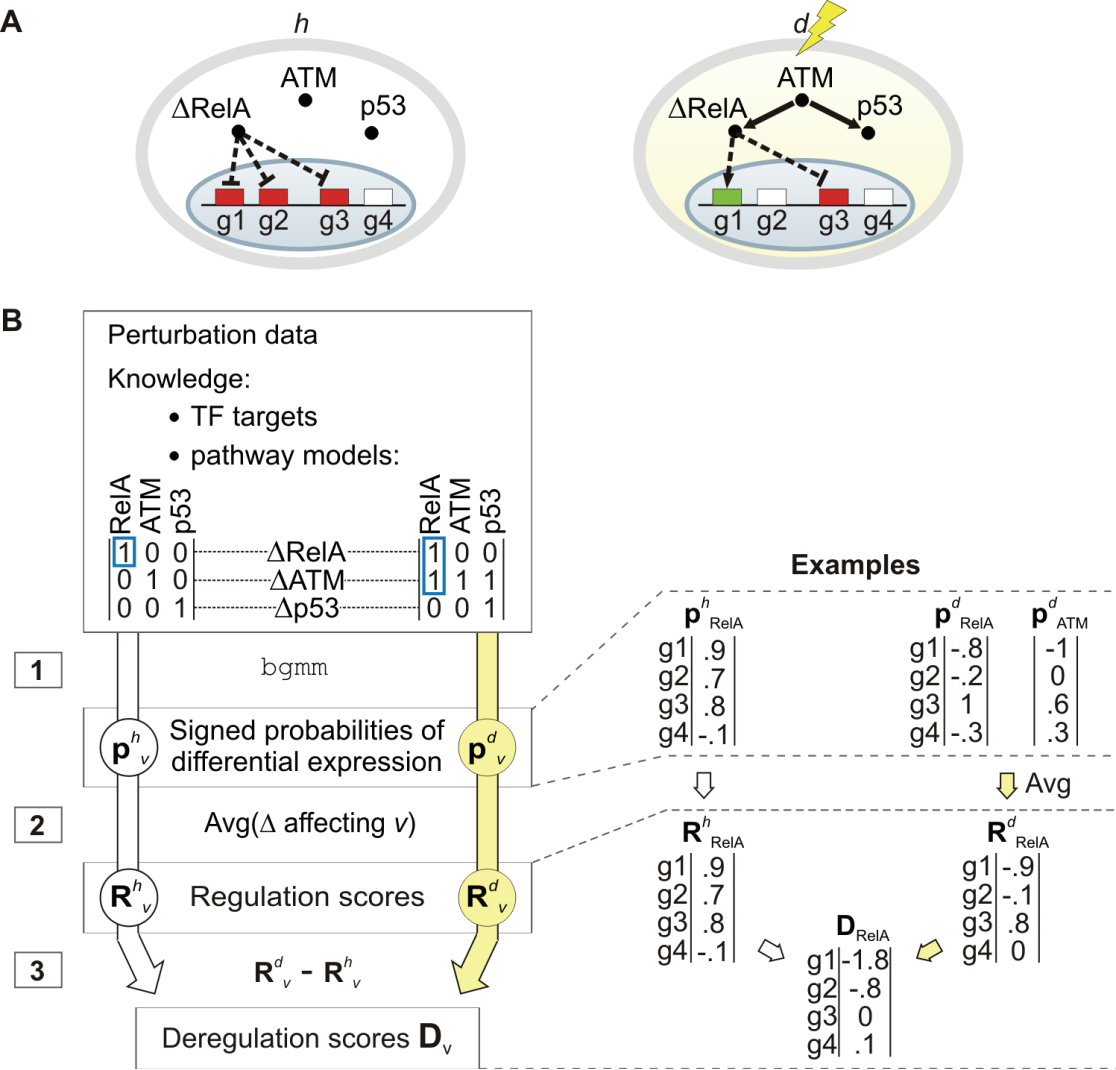
**Method overview**. (**A**) Two different cells (ovals): a healthy cell *h *in a neutral environment (left) and a damaged cell *d *treated with neocarzinostatin (right). Inside each oval: a pathway topology, with regulators RelA, ATM and p53, and a set of remaining genes *g*1-*g*4. Solid edges: signaling relations (e.g. in *d*, ATM signals down to RelA). Dashed edges: transcriptional regulation. Δ RelA - an experiment, where RelA is perturbed. Gene colors: effect of perturbation (up-regulation in red, down-regulation in green and no change in white). (**B**) The JODA algorithm. Input: (i) perturbation data, (ii) known TF-targets, and (iii) known pathway models encoded in matrices with an entry 1 when a perturbation experiment (rows) affects the regulator (columns; otherwise the entries are 0). Experiments affecting RelA are marked in blue. The input is processed for the healthy (left) and the damaged cells (right) separately in three steps, until merged in deregulation scores. Examples on the right illustrate the steps for RelA. First step: 'bgmm' [32] is applied to identify probabilities of differential expression of the genes under perturbation of each regulator *v *(denoted  and  for the two cell populations *h *and *d*). Each probability is multiplied by -1 when the effect of perturbation was down-regulation, or by +1 when the effect was up-regulation. Second step: we compute regulation scores  and , which quantify the effect of each regulator *v *on the genes in a given cell population. Third step: we subtract regulation scores in the healthy cells from regulation scores in the damaged cells to obtain deregulation scores **D***_v_*, quantifying how strongly each regulator *v *deregulates the genes.

In application to analysis of deregulation driven by DNA damage in Human cells, JODA reveals broad changes of gene regulatory network downstream of the ATM signaling pathway. The analysis integrates expression data from perturbation experiments in the healthy cells and in cells undergoing DNA damage [28] (see Figure 1A), the knowledge about ATM signaling down to RelA and p53 (absent in the healthy cells and active in the damaged cells), together with the known targets of RelA and p53 in both cell populations. The damaged cells are obtained by exposure to neocarzinostatin (NCS), an antibiotic that induces DNA double strand breaks and activates the ATM pathway [29-31]. Original data analysis [28] rigorously but exclusively focused on a small set of 112 genes responding to NCS treatment, which showed perturbation effects that correctly reconstructed the known ATM pathway interactions. Here, based on the deregulation scores, we cluster 645 genes into thirteen functional clusters, reflecting the rich spectrum of biological activities in the DNA damage response program. We review genes in the functional clusters in terms of the known impact of NCS on its gene targets. Analyzing enrichment in canonical pathways and known gene-regulatory and protein-protein interactions, we elucidate the connectivity within those functional clusters. We list genes with the most extreme deregulation scores reporting their involvement in DNA damage response. Our results validate that genes with dominant deregulation scores are directed by the ATM pathway and are functionally involved in the switch between the healthy and damaged cells induced by NCS. In the final section we show that the approach can also lead to testable hypotheses: we investigate the indirect regulatory impact of each ATM, RelA and p53 on the deregulated genes, and build a hypothetical hierarchy of direct regulation.

## Results

### A method for quantifying deregulation

JODA reveals deregulation between two different populations of cells. We distinguish two sets of genes: *regulators*, and all *remaining genes *(shortly, *genes*). The regulators are components of a signaling pathway, which is important for the switch between the cell populations, and which may have a different topology in one cell population than in the other. We require that each regulator is perturbed in both cell populations. The remaining genes show effects of the perturbations in their expression. We are interested in regulatory relations connecting regulators to the remaining genes and how these relations change between the cell populations.

In addition to perturbation data, for each cell population, JODA takes as input two kinds of qualitative knowledge. The first kind of knowledge are two *pathway topologies*, which describe the signaling relations between all regulators within the pathway in the two cell populations. The set of regulators involved in the two topologies is assumed to be the same, but the signaling relations can be different. The signaling relations describe 'who signals to whom' in both populations and may be derived from multiple sources: the researcher's expertise, literature findings, external experimental data or application of a reverse engineering method of choice. This knowledge is given to the input of JODA in a form of two directed graphs (one per each cell population). The nodes in the graphs correspond to the regulators (pathway components). There is an edge between two nodes in a given pathway topology whenever it is known that the pathway component corresponding to one node activates the component corresponding to the other node. The graphs may be cyclic and may have several connected components. Examples of two given ATM pathway topologies, one in the healthy cells (denoted *h*) and second in cells undergoing DNA damage (shortly, *damaged *cells, denoted *d*), are illustrated in Figure 1A.

Internally, based on the given pathway topologies, JODA builds two binary matrix models. The pathway models are used by the algorithm to determine which perturbation experiments affect which regulators in the pathways. For a given pathway topology graph, JODA first adds an edge going from each node to itself, which corresponds to the trivial fact that a perturbation of a given regulator influences this regulator. Next, JODA computes a transitive closure of the graph, which corresponds to predicting how the effects of the perturbation experiments propagate in the pathway. The pathway model is given by a matrix representation of the resulting graph. It contains an entry 1 whenever a perturbation of a regulator corresponding to the row affects the regulator corresponding to the column (otherwise the entries are 0). For each regulator, the model defines a set of perturbation experiments which affect this regulator's activity. See Methods for a formal introduction of the model. Example pathway models for the ATM pathway in the healthy and in the damaged cells are shown in Figure 1B.

The second kind of knowledge are regulator-gene relations, given for some regulators, which are also TFs, and for some remaining genes. This knowledge, similarly as pathway topologies, is cell-population specific and thus is given separately for each population. It originates either from the individual TF targets established in the literature, or from high-throughput TF DNA-binding data. The known TF targets are expected to show an effect to the perturbation experiments, and serve as examples of genes that are differentially expressed upon their TF perturbation. This kind of knowledge is rarely certain and in our approach is given as a belief about the TF-target relationships, rather than a fixed statement.

JODA processes given data and knowledge in three steps (Figure 1B). In the first step, we analyze the input data from each perturbation experiment to estimate the effect of the perturbation on the genes. To this end, we apply our belief-based differential expression analysis method (Methods), implemented in the R package 'bgmm' [32]. The method assigns each gene a probability that it was differentially expressed in the experiment. In this step, the knowledge about the known TF targets is used. To improve the estimation, the known targets of the perturbed regulator are given a high prior of differential expression in the perturbation experiment. We multiply each returned probability by +1 or -1 to indicate whether the effect of the perturbation was up- or down-regulation, respectively. Thus, each signed probability lies in the [-1, 1] interval. For a given perturbation of regulator *v *in cell population *t*, the vector of signed differential expression probabilities of the genes in this experiment is denoted .

In the second step, for each regulator *v *and cell population *t*, we obtain a vector  of *regulation scores *that quantify the effect of *v *on the genes in *t*. In this step, the knowledge about the pathway topologies is used. For a given cell population and regulator, regulation scores are computed as an average over the signed probabilities of differential expression in all perturbation experiments that affect this regulator in this cell population. Using the pathway model, the affecting experiments are defined as both the perturbation of the regulator itself, and perturbations of its upstream activators in the pathway (Methods). For example, in Figure 1B the regulation scores  for RelA in the damaged cells are an average of signed probabilities for the perturbations of RelA and of its upstream activator ATM. In the healthy cells, only its own perturbation affects RelA, and its regulation scores  are the same as its signed probabilities . Assuming that the model is correct, the experiments affecting a given regulator should have a common effect on this regulator's target genes. In other words, each target gene is expected to have either high or low signed probabilities of differential expression that are consistent between all affecting experiments. Thus, taking an average yields either high or low regulation scores for the true targets, and rules out those genes which respond to the perturbation experiments in a model-independent manner. A negative regulation score indicates (possibly indirect) activation of a gene, and a positive score indicates inhibition. This rule, counter-intuitive at first sight, is motivated by the fact that genes with positive regulation scores have mostly positive probabilities of differential expression, i.e., tend to be up-regulated in those perturbation experiments that affect their regulator. The genes with negative scores have mostly negative probabilities, i.e., are down-regulated. Accordingly, we define genes *more activated *in a given cell population (e.g., damaged *d*), as having lower regulation score in this cell population than in the other (e.g., healthy *h*). For example, in Figure 1*g*1 is up-regulated upon the RelA perturbation (possibly indirectly inhibited by RelA) in *h *and is down-regulated (possibly indirectly activated by RelA) in *d*. *g*2 is up-regulated upon the perturbation in *h *and shows no effect in *d*. Thus, genes *g*1 and *g*2 are more activated in *d*.

In the third step, to quantify deregulation of genes by a given regulator *v*, we define a vector **D***_v _*of *deregulation scores *as the difference between the regulation scores for *v *in the two cell populations. In this way, each deregulation score lies in the [-2; 2] interval. For example, in Figure 1 genes *g*1 and *g*2 are deregulated between the healthy and damaged cells, while gene *g*3 stays regulated the same way, and *g*4 is not dependent on the pathway. Accordingly, *g*1 and *g*2 have dominant deregulation scores, which are well discriminated from the scores of *g*3 and *g*4 (Figure 1B). Note that in the case when regulation scores for cell population *h *are subtracted from scores for cell population *d*, genes more activated in *d *(e.g., genes *g*1 and *g*2 in Figure 1) obtain negative deregulation scores, whereas genes more activated in *h *obtain positive scores.

### Deregulated genes identified by JODA group into biologically relevant functional clusters

JODA was applied to identify genes deregulated in response to DNA damage induced by NCS, a drug known to cause double strand breaks in the DNA [29]. We analyzed transcriptional effects of silencing the regulators ATM, RelA and p53, performed by Elkon *et al*. [28] on the healthy and the damaged cells (together six perturbation experiments). The data for each perturbation experiment are log expression ratios of a regulator knockdown versus control in a given cell population. See Methods for data processing. Additionally, we provided two kinds of knowledge. First, the ATM pathway topologies in the damaged and in the healthy cells. As presented in Figure 1A, in the damaged cells NCS triggers a cellular pathway, where the central kinase ATM signals down to TFs RelA and p53 [30]. This pathway is inactive in the healthy cells. Second, we provided known targets of RelA and p53 in the two cell populations (listed in Additional file 1; see Methods).

Application of our approach resulted in three lists of deregulation scores (shortly, *deregulation lists*), one for each of the regulators ATM, RelA and p53. We sorted the deregulation lists decreasingly, so that the one extreme of each list contains genes more activated in the healthy cells, and the other contains genes more activated in the damaged cells. We performed Gene Set Enrichment Analysis (GSEA [33]; see Methods) to identify gene sets significantly overrepresented on the extremes of the lists. We focused the analysis first on the enrichment of GO terms (taken from the MSigDB database [33]).

**Figure 2:**
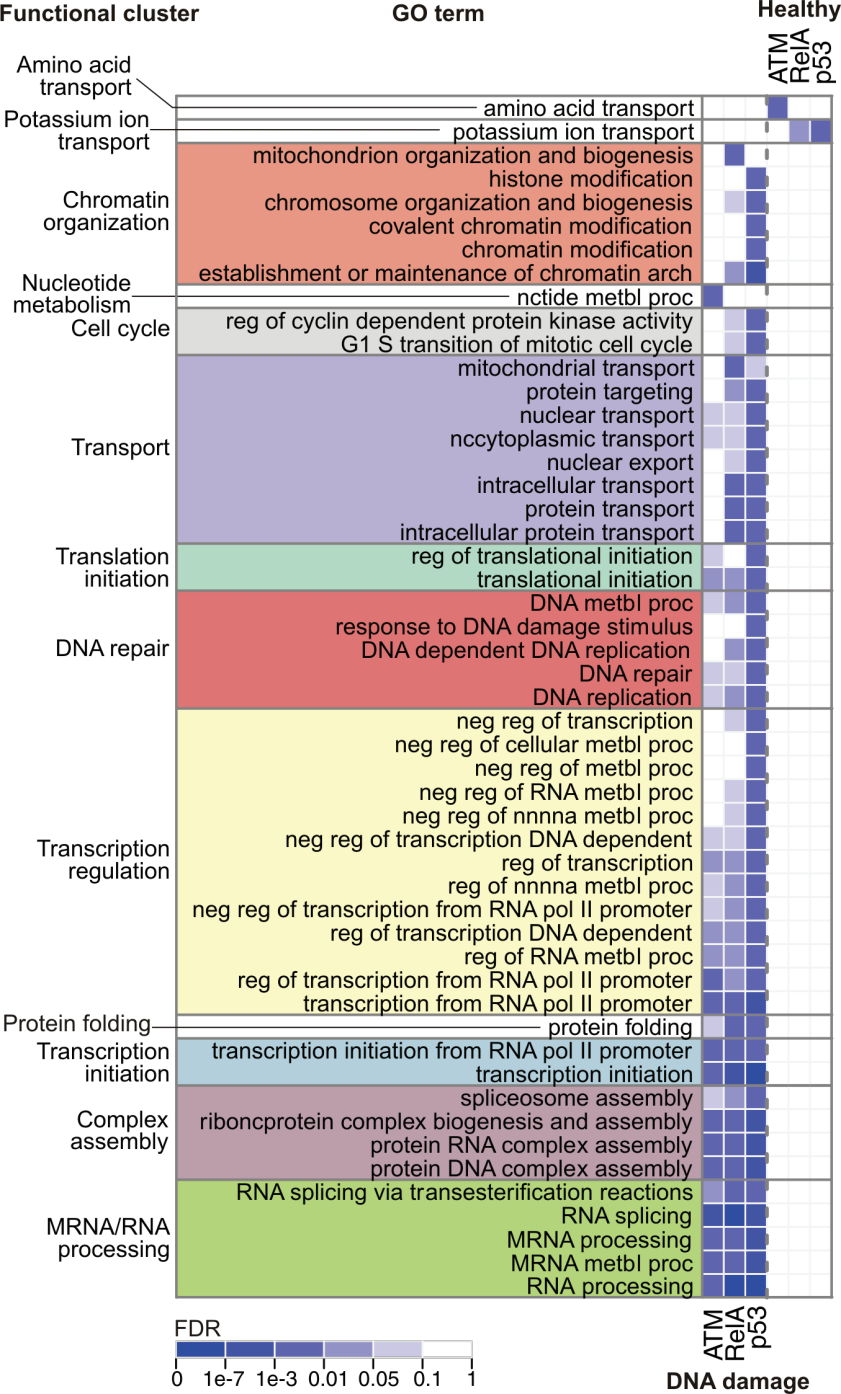
**Functional enrichment**. The matrix shows GO terms enriched with high confidence (FDR ≤ 0.01, indicated in blue, and FWER ≤ 0.5; identified using GSEA [33]) in the genes more activated in the damaged cells by ATM, RelA and p53 (left three columns) and in the genes more activated in the healthy cells (right three columns). Each GO term shown is enriched in at least one column. The terms were grouped into functional clusters with names indicated on the left (Methods), and sorted by the average enrichment in the first three columns. The GO term enrichment is mutually exclusive for the genes more activated in the healthy and in the damaged cells. Eleven functional clusters of GO terms are enriched exclusively in genes more activated in the damaged, and two exclusively in the healthy cells. Abbreviations: mtbl, metabolic; nc, nucleo; pol, polymerase; reg, regulation; neg, negative; pos, positive; proc, process; arch, architecture; nnnna, nucleobase, nucleoside, nucleotide, and nucleic acid. The identified clusters confirm that the dominant deregulation scores are correlated with a functionality which is highly relevant to the switch between the compared cell populations.

Figure 2 presents 51 identified overrepresented GO terms and their enrichment in the deregulation lists. The terms were grouped by hierarchical clustering according to the 'relevance' similarity measure [34] into thirteen *functional clusters*, where each cluster is labeled with a common general biological function (see Methods). Several functional clusters, e.g. *DNA repair*, *Chromatin organization*, *Transcriptional regulation *and *Cell cycle*, indicate that our method assigns dominant deregulation scores to genes playing crucial roles in response to DNA damage. Moreover, we find enrichment of deregulated genes in *mRNA/RNA *and *Nucleotide processing*, *Complex assembly*, *Protein folding*, *Transport *as well as transcription- and translation-related processes. This rich involvement of genes up-regulated in response to DNA damage in various processes is in agreement with previous findings [35,36].

Eleven clusters of GO terms are found for the genes more activated in the damaged cells and only two in the healthy cells, even though the distributions of the deregulation scores have the median of zero and are not biased in number towards the negative values (Additional file 2, Figure S1). The eleven clusters more activated in the damaged cells are shortly referred to as *damage-activated*, and the two more activated in the healthy cells are called *healthy-activated *throughout the text. Strikingly, the regulators agree on the functional processes they activate: no GO term overrepresented in the genes more activated in the damaged cells is also overrepresented in the genes more activated in the healthy cells. This shows the tightly coordinated way in which the ATM pathway governs the downstream response to the damaging agent. From the functional clusters of GO terms we identified clusters of deregulated genes, which reside on the extremes of the deregulation lists, and are annotated with those terms (Methods). As a result, 645 genes are separated into thirteen functional gene clusters of different sizes (Additional file 2, Figure S2). The number of clusters was chosen to maximize the ratio of the number of clusters over the number of genes shared between the clusters. This choice is a tradeoff between maximizing the diversity of functions in the clustering and minimizing the overlap between the clusters (Additional file 2, Figure S3). The main general function of each gene cluster is captured by its label. To annotate clusters with additional, secondary functions we used the Ingenuity Pathway Analysis software (Ingenuity Systems). Additional file 3 lists all Ingenuity functions that are significantly overrepresented in each of the five largest clusters. Importantly, enrichment analysis of the *DNA repair*, *Transcription regulation *and *Chromatin organization *clusters revealed that they also contribute to *cell death*, *cell cycle*, as well as *cellular growth *and *DNA replication, recombination, and repair*. These three clusters are also significantly enriched in cancer-related genes. All three have strong enrichment for tumorigenesis processes, leukemia-related genes, as well as other cancer types (Additional file 4), which agrees with the known connection between DNA damage and cancer [37]. To address the quality of the deregulation scores, we review the genes in the deregulated functional clusters in terms of knowledge about the impact of NCS treatment on the genes in the treated cell. Using Ingenuity, we collected a set of 27 genes which are directly or indirectly influenced by NCS.

Additional file 2, Figure S4 presents what is known about the type of action of NCS on the targeted genes, as well as what is known about the NCS *not *acting on the genes. In majority of cases, this knowledge is consistent with the presence or absence of genes in the functional clusters. There is a significant overlap between the NCS-targeted genes and the deregulated functional clusters (hypergeometric higher tail *p*-value 7.75 e-05). These results confirm that our findings are consistent with the current knowledge about the action of NCS and expand it to the level of transcriptional and regulatory switch between the two analyzed cell populations.

### The deregulated functional clusters and pathways cannot be found without prior knowledge or in separate analysis

The power of JODA becomes apparent when comparing it to two simpler methods. First, an analysis without incorporation of prior knowledge, which is based only on taking differences of correlation (*decorrelation*) of expression between the regulators and the remaining genes. Second, to a separate analysis of the two cell populations, which is based on inferring regulation in the two compared populations of cells separately, and only then finding the differences. The grounds for the comparison of JODA to the two simpler methods is the significance of scores assigned to genes with key functions that are expected to be deregulated between the two compared populations.

To analyze decorrelation, for each analyzed gene and each regulator (ATM, RelA, and p53) we computed Pearson correlation first between the expression profiles of the regulator and the gene measured in the healthy cells, and second between the profiles of the regulator and the gene in the damaged cells. Strong positive correlation in a given cell population can be interpreted as an activation of the gene by the regulator in this cell population, whereas strong negative correlation can be interpreted as inhibition. To obtain the decorrelation scores for each regulator, we subtracted the correlation values for all genes in the damaged cells from the correlations in the healthy cells. In this way, the decorrelation scores, belonging to the interval [-2, 2], can be read similarly as the deregulation scores: strongly negative decorrelation scores indicate more activation in the damaged cells, and strongly positive indicate more activation in the healthy cells. The decorrelation scores are a simple implementation of the ideas applied by Taylor *et al*. [14] and Mani *et al*. [13]. Taylor *et al*. [14] used Pearson correlation of interactome hubs to their interaction partners to verify whether these interactions are context-specific. Mani *et al*. [13] investigated gain and loss of correlation between cell populations using a mutual information-based approach. Although interpreted in the same way, the decorrelation scores differ from the deregulation scores in two important ways. First, they do not incorporate given prior knowledge about the known cell population-specific pathway topology nor target genes downstream of the pathway. Second, they measure the activity of the regulators (which are proteins) by their expression levels, ignoring the fact that it is modulated on post-translational level. To perform the separate analysis, we analyze the regulation scores. Regulation scores are returned from the second step of JODA (Figure 1B), separately for each cell population. Thus, in contrast to the decorrelation scores, they are obtained using both types of knowledge given as input to the algorithm. Recall that in a given cell population, extreme regulation scores are given to those genes which according to the perturbation data and knowledge are directly or indirectly controlled by the regulators.

**Figure 3:**
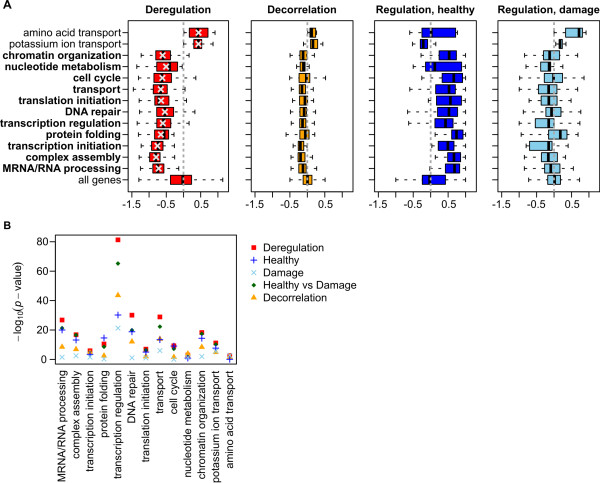
**Functional gene clusters are significantly deregulated**. (**A**) Distributions of the deregulation scores of the genes in the functional clusters (averaged over the three regulators, ATM, RelA and p53) strongly deviate from the distribution of averaged deregulation scores for all genes (left plot). The distributions of average decorrelation scores (middle left), as well as of average regulation scores in the healthy (middle right) and in the damaged cells (right), are more similar to the distributions of the same scores for all genes. Gray dashed vertical lines mark score 0 in each plot. The names of the eleven damage-activated clusters are in bold. (**B**) A *t*-test comparing the cluster deregulation scores with the deregulation scores for all genes (Deregulation; red squares) gives for majority of the clusters the most significant *p*-values, when contrasted with: the *p*-values obtained in a *t*-test comparing cluster regulation scores to regulation scores of all genes in the healthy cells (Regulation, Healthy; blue pluses), and the same *t*-test but in the damaged cells (Regulation, Damage; light blue crosses), the *p*-values in a *t*-test comparing cluster regulation scores in the healthy directly to regulation scores in the damaged cells (Healthy vs Damage; green diamonds), and in a *t*-test comparing the cluster decorrelation scores with the decorrelation scores for all genes (Decorrelation; yellow triangles). All tests are two-sided. Taken together, our joint and knowledge-based approach assigns more significant scores to the functional clusters than a separate analysis, or an analysis without incorporation of knowledge.

JODA outperforms the analysis performed with the decorrelation scores. Figure 3 shows that the genes in functional clusters have deregulation scores that stand out significantly from the background of deregulation scores for all analyzed genes. Using the decorrelation scores, the same two clusters can be identified as healthy-activated and eleven as damage-activated, but they are less significant than when deregulation scores are used. Similarly, several clusters, although performing functions important for the switch between the healthy and damaged cells, are likely to be missed when analyzing the cell populations separately. For example, based on the regulation scores in the damaged cells only, the genes in the *DNA repair *cluster cannot be significantly differentiated from all genes (Figure 3B).

To further compare the joint approach to the separate analysis, we applied GSEA to perform GO term enrichment analysis in the sorted lists of regulation scores. Such a list contains on the one end the genes which are up-regulated by the regulator perturbation (i.e., are possibly indirectly inhibited by the regulator) and on the other the genes that are down-regulated by the perturbation (indirectly activated by the regulator). Additional file 2, Figure S5 compares GO terms or pathways overrepresented on the extremes of deregulation lists with the terms/pathways overrepresented on the extremes of sorted lists of regulation scores. First, the separate analysis misses gene sets that are only slightly down-regulated in one cell population and slightly up-regulated in the other. Deregulation scores, being a difference of the small but opposing effects, amplify them, making detection of such gene sets possible. For example, significant enrichment of the GO term *regulation of transcription *is only found by our approach and not by the separate analysis (Additional file 2, Figure S5 A). Second, the separate analysis identifies also GO terms or pathways annotating genes that show the same effect to perturbation in both cell populations, e.g. the glycolysis pathway, which is up-regulated upon knockdown of RelA and ATM both in the healthy and in the damaged cells (Additional file 2, Figure S5 B). Importantly, such effects do not characterize the difference between the two cell populations and in our analysis automatically cancel out when computing the deregulation scores. Taken together, deregulation scores are more sensitive to and oriented on differences between the cell populations (Additional file 2, Figure S5 C).

### Deregulated pathways and complexes elucidate cooperation within the functional clusters

**Figure 4:**
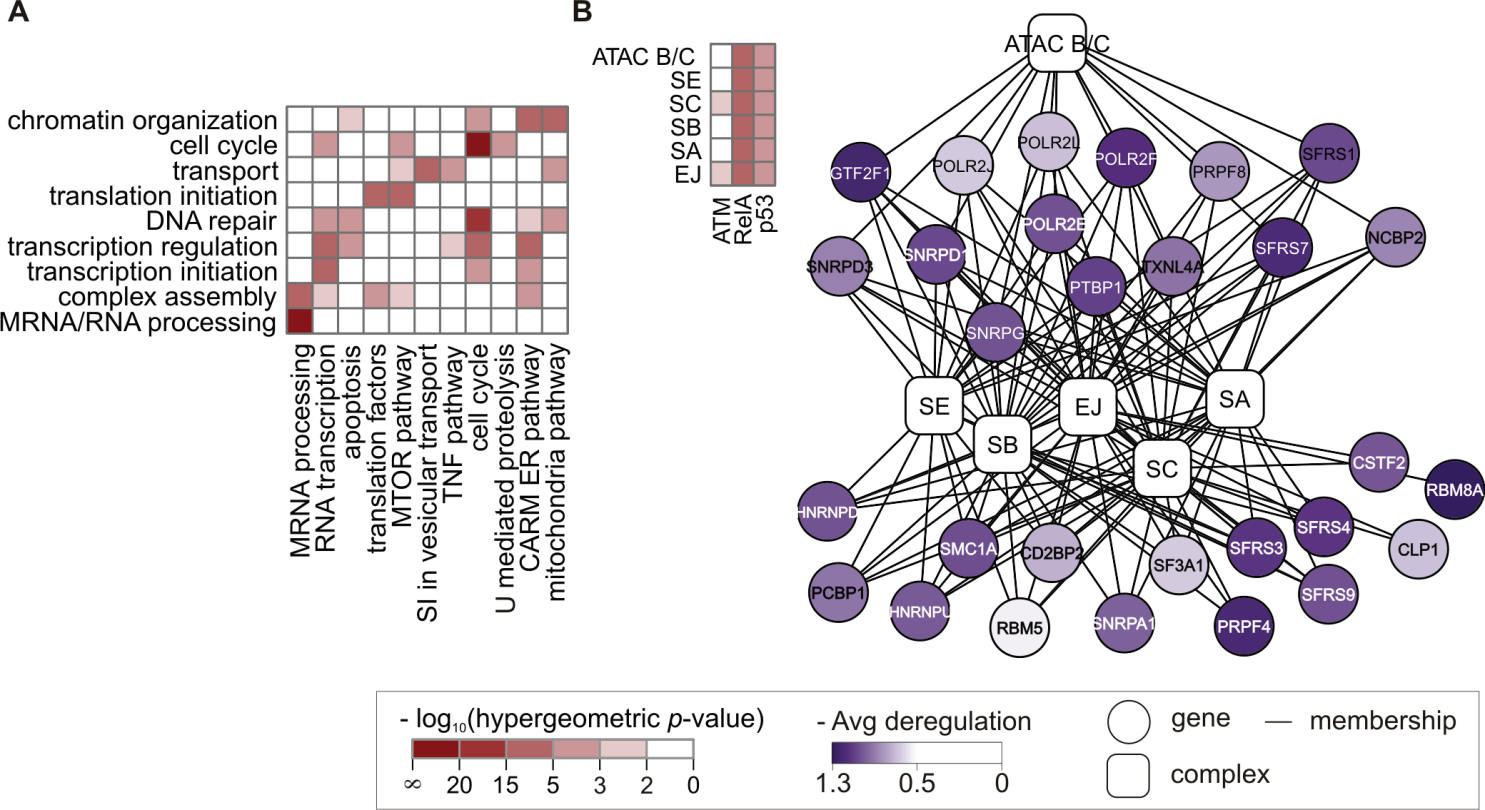
**Connectivity between genes in the functional clusters**. (**A**) Deregulated pathways. Matrix shows pathways (columns) enriched in the genes more activated in the damaged cells that overlap significantly with functional clusters (rows). Only pathways and clusters overlapping with higher-tail hyper-geometric *p*-value at most 0.001 (indicated in red) are reported. Abbreviations of pathway names: SI, snare interactions; U, ubiquitin. (**B**). Deregulated complexes. Top left: The Exon junction (EJ) complex and five spliceosome complexes (rows) are overrepresented (best hyper test, see Methods) in the genes more activated in DNA-damage by the regulators ATM, RelA and p53 (columns). Only complexes with a *p*-value at most 10*^-^*^5 ^(indicated in red) are reported. Genes in those complexes overlap significantly with the functional cluster *MRNA/RNA processing*. Right: Graph representation of the genes (round nodes shaded in violet by their average deregulation scores) in the complexes (rounded square nodes). Edges represent gene - complex membership. Abbreviations are reported in Additional file 2, Table S1. Pathways and complexes carry information about relations between their member genes. Therefore, these enrichment results broaden our view on the connectivity within the sets of genes in the functional clusters.

Since the genes in each cluster share the same functionality, they may directly interact in a common cellular pathway or complex. To determine these interactions we first identified pathways and complexes that are overrepresented on the extremes of the deregulation lists. Next, we checked their overlap with the functional clusters. The enrichment in pathways, similarly as for GO terms, was assessed using GSEA. The identified pathways are stored in the MSigDB database as sets of genes, but their signaling relations are well described in the literature. Eleven MSigDB pathways that are overrepresented on the extremes of the deregulation lists significantly overlap with our functional clusters (Figure 4A). For example, the *apoptosis *pathway contains genes from the *DNA repair *and *Transcription regulation *clusters. Moreover, we found the *Exon junction *complex and several spliceosome complexes (Figure 4B) significantly overrepresented in the genes more activated in the damaged cells (best hyper test, see Methods). Interestingly, these complexes overlap (hyper-geometric higher tail *p*-value 1.1·10 ^-29^) with the *MRNA processing *cluster. Similarly as pathway interactions, membership in complexes explains the way the genes in the clusters are interconnected and collaborate to exhibit the common function.

**Figure 5:**
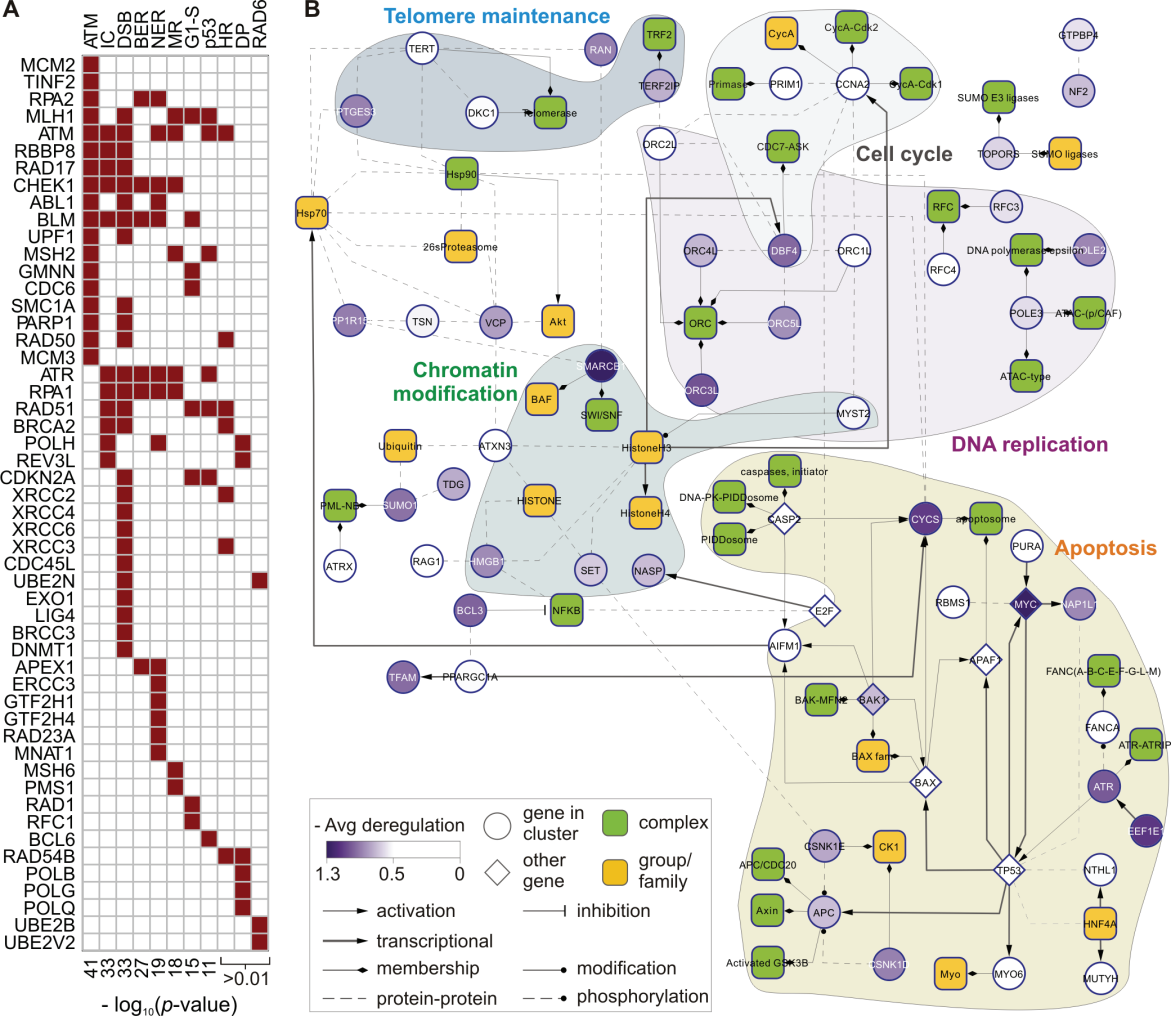
**Cooperation of the genes in the DNA repair cluster**. (**A**) The matrix shows 51 out of 117 genes in the *DNA repair *cluster, which belong (marked in red) to eleven known pathways involved in DNA repair (columns; listed on the top). First eight of those pathways are strongly enriched in the cluster (identified using SPIKE [38]). Three other patways (reviewed by Wood *et al*. [39]) overlap with the cluster, but not significantly (*p*-values are listed on the bottom). Abbreviations: ATM, ATM pathway; IC, repair of interstrand crosslinks; DSB, repair of double strand breaks; BER, base excision repair; NER, nucleotide excision repair; MR, mismatch repair; G1-S, G1-S pathway; p53, p53 pathway; HR, homologous recombination; PD, polymerase; RAD6, RAD6 pathway. Such strong enrichment in canonical pathways confirms the biological relevance of the deregulated genes in the *DNA repair *cluster. (**B**) To identify interconnections between the remaining 66 genes in the cluster, we searched for pathways of length at most one connecting each pair of those genes in a protein-protein and protein-DNA interaction network (using SPIKE and Ingenuity). The resulting graph connects 33 genes (remaining 33 are isolated and not displayed) and represents the complexes to which the genes belong. Some of the complexes are involved in a common process: DNA replication, apoptosis, cell cycle, or telomere maintenance. The network explains connectivity within the *DNA repair *cluster that goes beyond the canonical pathways.

Finally, we focused on the *DNA repair *cluster, which is of pivotal interest in the context of the switch between the healthy and the damaged cells. We investigated physical relations among genes within this cluster. The cluster is strongly enriched in eight pathways involved in response to DNA damage (*p-*values from 1.33·10^-41 ^to 8.17·10^-11^; identified using SPIKE [38]). SPIKE is a database and an analysis tool, storing manually curated pathways, which play key roles in response to damage. The table in Figure 5A lists 51 genes from the *DNA repair *cluster, which belong to those canonical pathways stored in SPIKE as well as three additional pathways, described in a comprehensive review on DNA damage response by Wood *et al*. [39]. The significantly enriched pathways include non-homologous end-joining and homologous recombination, which are typical pathways responsive to DNA double strand breaks [40,41]. The position of the listed genes in the well known damage response pathways describes their role in the response, as well as their interaction partners in the cluster. To further infer the cooperation between the remaining genes in the *DNA repair *cluster we collected their interactions using SPIKE and Ingenuity (Figure 5B; see Methods). The analysis revealed a number of complexes that join subsets of genes together, e.g. the Origin of Replication complex (ORC) containing five *DNA repair *genes. Grouping the complexes by common functionality, we selected functional sub-parts of the network. For example, we identified a sub-network of genes belonging to the RFC, DNA polimerase epsilon, and the ORC complexes, which are involved in the DNA replication process (marked with a light grey background in Figure 5B).

### Genes most activated in damaged cells function in the ATM pathway-induced damage response

Functional clusters contain deregulated genes that accumulate within the extremes of the deregulation lists, but not on the strict top or bottom. We investigated the composition of three sets of one hundred genes that are most activated in the damaged cells by each regulator RelA, ATM and p53 (Additional file 5A). All three sets are significantly enriched in genes involved in *transcription*, with five common genes active in this process: *CHD4*, *RBM14*, *RCAN1*, *SMAD4*, and *UBN1*. Interestingly, some of the genes most activated by RelA are interaction partners (for example, *SMARCB1*) of the genes most activated by p53 (*SMARCB4*). Apart from *transcription*, the set most activated by ATM is also enriched in *cell death*, *cell cycle*, and *growth*-related genes. Moreover, both sets of genes most activated by ATM and p53 are enriched in cancer-related genes (Additional file 5B).

The stability of deregulation scores for the genes most activated by any of the three regulators (ATM, RelA, and p53) was assessed based on *p-*values for a permutation test. Deregulation scores measure the relative change of probability that a gene is up- or down-regulated. The permutation test was used to verify a hypothesis that for a given gene its deregulation score is significantly different than zero. To perform the test, the signed probabilities of differential expression for all genes and regulators were permuted at random 1000 times. The *p*-value for the permutation test was calculated as a probability that the observed deregulation score is closer to zero than the deregulation scores calculated for the permuted differential expression probabilities. There are two benefits of permuting the differential expression probabilities (returned from the first step of JODA) instead of the input expression dataset. First, we directly test the effect of the pathway models on the deregulation scores (since the pathway models are incorporated in the second step). Second, we avoid fitting Gaussian mixtures to the data based on beliefs with random observations. Additional file 2, Figure S6 presents the distribution of *p-*values for the most activated genes versus all other genes. All genes most activated by RelA and p53 have *p*-values lower than 0.05. Genes most activated by ATM have less significant scores.

**Figure 6:**
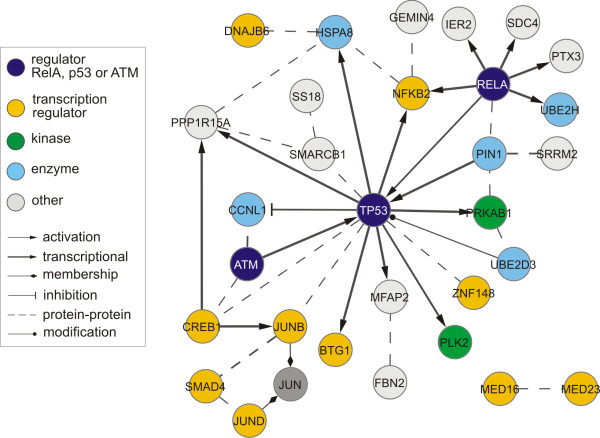
**A network of interactions between genes most activated in DNA damage**. A network of known relations (edges) connecting 28 genes (nodes) from the three lists of top twenty most activated by RelA, ATM or p53 in DNA damage. The relations are collected from the Ingenuity Pathway Analysis (Ingenuity Systems) database. The nodes are labeled with gene names and colored according to gene functions, whereas relations are given edge styles according to their type.

Next, we reviewed the individual examples out of three shorter lists of twenty genes that are most activated in the damaged cells by RelA, ATM and p53. Those shorter lists contain together 51 unique genes (see Additional file 6 for a detailed list of their regulatory relations, collected in Ingenuity). Figure 6A presents a network interconnecting 28 of the 51 genes, for which regulatory interconnections are known. Both p53 and RelA, with seven and five regulatory targets, respectively, are major regulators for the genes in this network. Moreover, 10 out of the 28 genes in the network are transcription regulators themselves. From all 51 most activated genes there are 12 transcription regulators, 19 genes involved in apoptosis, 18 in proliferation and 6 in cell cycle progression.

Additional file 2, Figure S7 A visualizes expression profiles of the most activated genes. Expression data explains the deregulation *p-*values showing that those genes tend to be repressed in the healthy, and activated in the damaged cells.

### RelA and p53 are the key deregulators of genes in functional clusters

Deregulation is inferred from perturbation effects, and as such can be due to an indirect impact of the regulators on the genes. Here, we summarize these possibly indirect effects on functional clusters of the deregulated genes identified by JODA.

**Figure 7:**
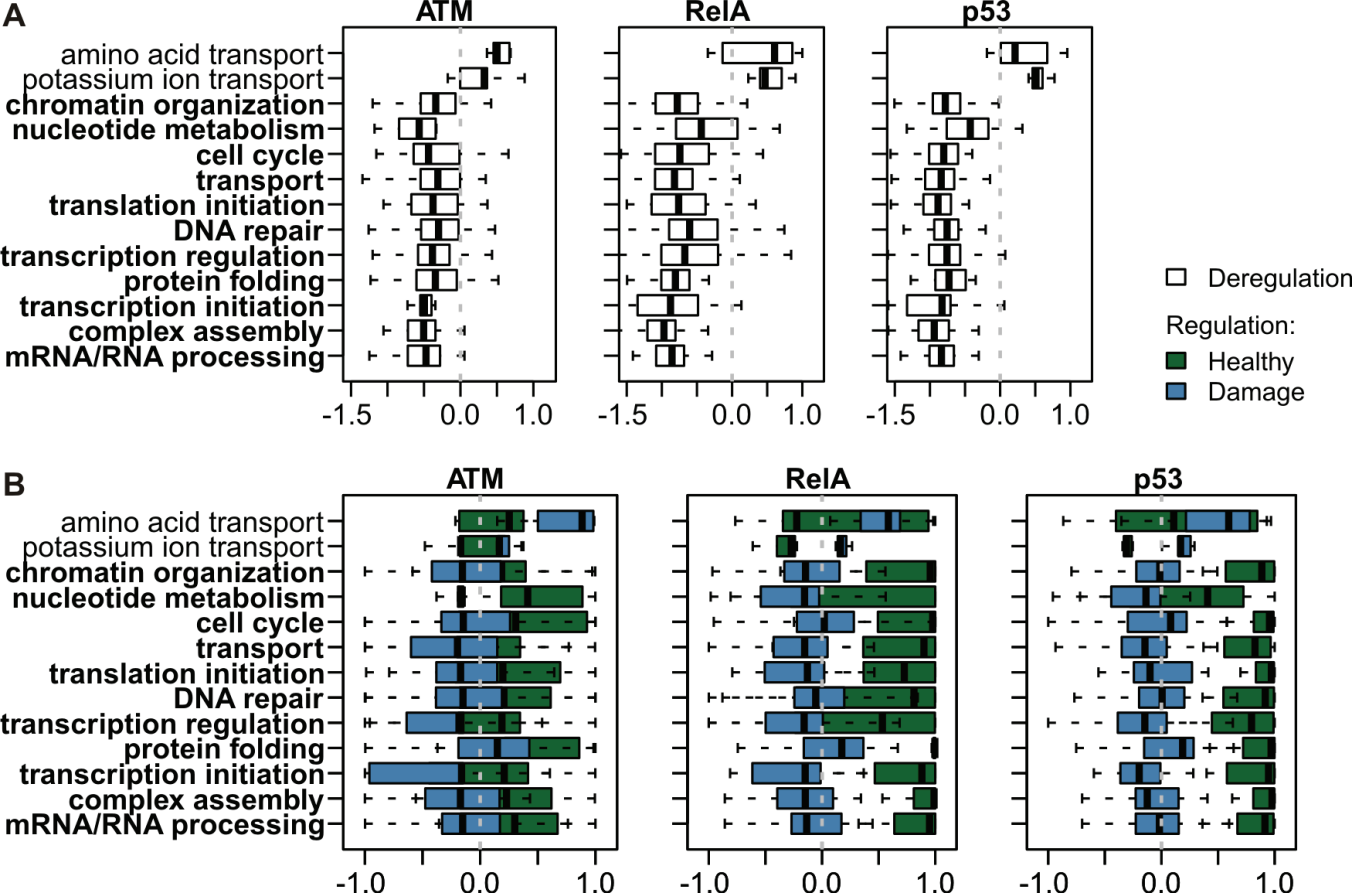
**Summary of indirect deregulation and regulation of each functional cluster**. (**A**) Distributions of the deregulation scores (*x*-axis) of the genes in the functional clusters (*y*-axis). (**B**) Distributions of the regulation scores (*x*-axis) in the healthy cells (drawn in green) and in the damaged cells (drawn in blue) of the genes in the functional clusters (*y*-axis). In **A **and **B **the score distributions are plotted separately for the three regulators, from left to right: ATM, RelA and p53. The names of the eleven damage-activated clusters are in bold.

Figure 7 reports deregulation and regulation scores of the genes in functional clusters, for each regulator ATM, RelA and p53. The per cluster distributions of deregulation scores for RelA and p53 are shifted further away from zero than for ATM, suggesting a stronger deregulatory impact on the clusters (Figure 7A). Indeed, Figure 7B shows that the distributions of regulation scores for ATM in the healthy and in the damaged cells are generally less separated than for RelA and for p53. Interestingly, for all three regulators, the regulation scores indicate that the damage-activated clusters are only slightly (possibly indirectly) activated in the damaged cells. Instead, these clusters are strongly (possibly indirectly) inhibited in the healthy cells both by RelA and by p53, as indicated by the respective distributions of regulation scores shifted towards value 1. The inhibitory impact of ATM on these clusters in the healthy cells is less prominent. In the case of the two healthy-activated clusters, a strong, possibly indirect inhibition in the damaged cells is observed for all three regulators. Distinctively, the *Potassium ion transport *cluster is also (possibly indirectly) activated in the healthy cells by RelA and p53.

### Deregulation can be explained by a hierarchy of direct TF-DNA binding events

Finally, we investigate the hierarchy of direct regulatory relations, which could explain the effect of the ATM pathway on the deregulated target genes. The first possible scenario would involve regulation by direct binding of the regulators in the pathway to the gene promoters. Alternatively, the most parsimonious hierarchy would connect the regulators to the genes via a single TF. To investigate these hypotheses, we follow a two step procedure. In the first step we computationally predict the TFs directly binding to the promoters of the genes. In the second step we verify whether the TFs are the regulators themselves, or whether they are controlled by the regulators.

**Figure 8:**
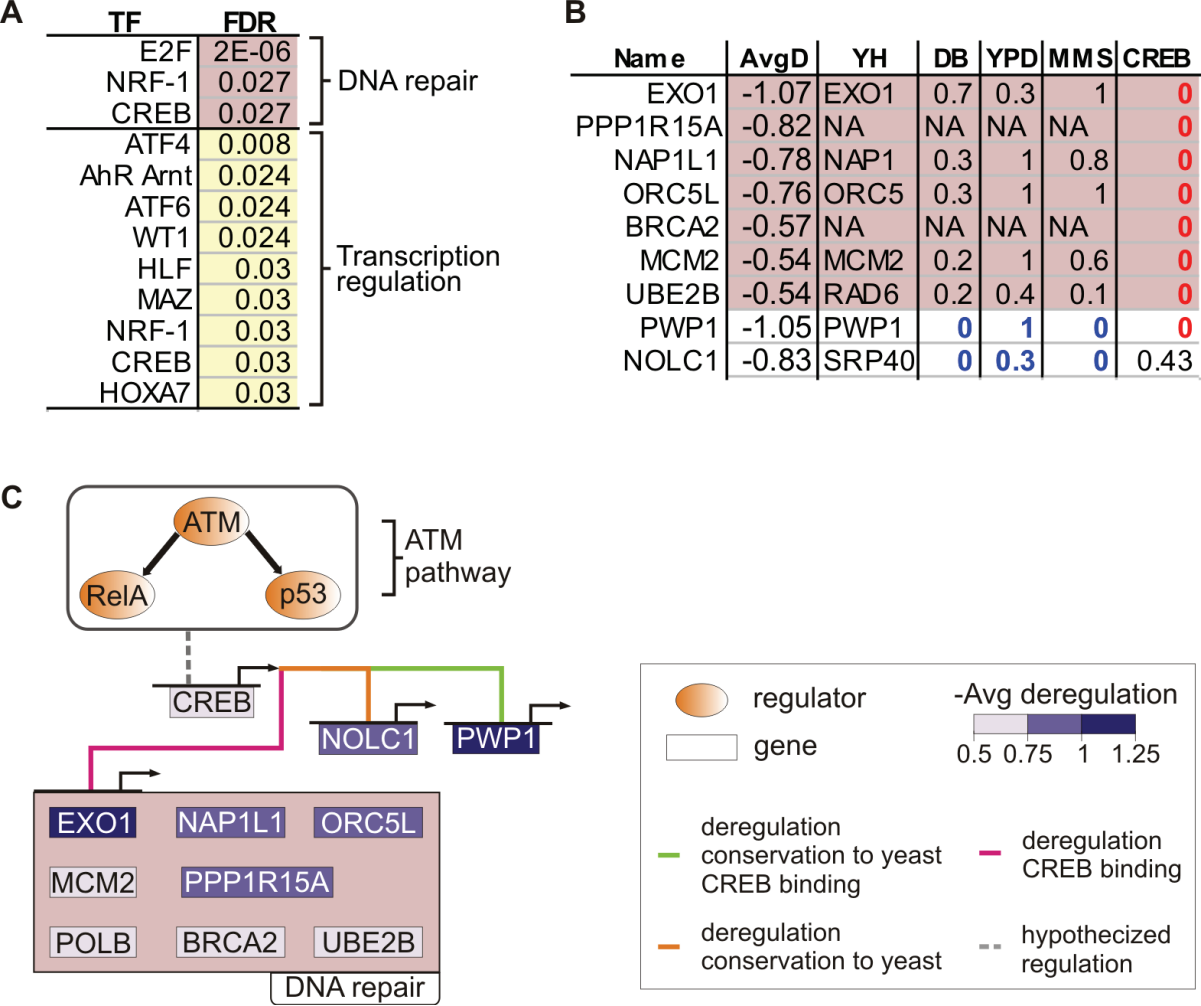
**A hypothetical deregulatory hierarchy**. (**A**) TFs with high affinity binding overrepresented in the promoters of genes in the functional clusters and in promoters of their mouse orthologs. Only binding predictions with FDR ≤ 0.05 are shown. (**B**) Nine deregulated genes with strong evidence of regulation by CREB. Column titles: Name, gene names; AvgD deregulation scores averaged over RelA, ATM and p53; YH, yeast homologs (NA - homolog not known); DB, *p*-values for deletion-buffering of the homologs by SKO1, a CREB homolog in yeast. YPD, *p*-values for the binding of SKO1 to the promotors of the yeast homologs in YPD medium; MMS, *p*-values for SKO1 binding to the promoters of the homologs in cells damaged by MMS. CREB, *p*-values for CREB binding to promoter, averaged over three time points of forskolin stimulation in HEK293T cells. Colored background marks genes from the *DNA repair *cluster that have a high affinity binding of CREB to their promoters conserved in mouse. (**C**) Putative gene deregulatory network. Top: ATM pathway. Middle: The pathway deregulating CREB. Below: CREB regulating its most likely gene targets (the genes shown in **B**). Genes are colored in shades of violet according to their deregulation, averaged over the regulators RelA, ATM and p53. The hierarchy is a hypothetical mechanistic explanation of deregulation of those genes, observed between the healthy and the damaged cells.

To implement the first step, we applied TransFind [42] to predict TFs binding to the promoters of the genes in each functional cluster (Figure 8A; see Methods). Among the identified TFs, CREB has binding sites significantly enriched in the promoters of genes in the *DNA repair *cluster. Neither RelA nor p53 were predicted to bind directly to the promoters of the genes in the functional clusters. Thus, in the second step we consider the hypothesis of the parsimonious hierarchy. Here, we focus on CREB, leaving other predicted TFs as candidates for future investigation. The hypothesis consists of a deregulatory connection from the ATM pathway to CREB, implemented by RelA or p53 directly binding to CREB promoter in the damaged cells and not binding in the healthy cells.

To complete the picture, based on several different criteria we collected nine most likely direct target genes of CREB, which are also deregulated in our system (Figure 8B). *EXO1*, *PPP1R15A*, *NAP1L1*, *ORC5L*, *BRCA2*, *MCM2 *and *UBE2B *belong to the *DNA repair *cluster and have a high affinity binding of CREB to their promoters conserved in mouse. Additionally, we report two genes outside of the cluster, *PWP1 *and *NOLC1*. Both are deregulated and have yeast homologs, which were identified by Workman *et al*. [12] to be *deletion-buffered *by SKO1, an yeast homolog of CREB. That means, both their homologs change expression in wild-type cells in response to methyl-methanesulfonate (shortly, MMS, a double-strand breaks-inducing drug) and do not change when SKO1 is perturbed. Moreover, the promoters of *PWP1 *and *NOLC1 *yeast homologs are not bound by SKO1 in the healthy yeast cells, and are bound by SKO1 in the cells damaged by MMS. The data of Zhang *et al*. [43] supports that the promoters of all nine target genes but *NOLC1 *are bound by CREB in HEK293T cells.

Figure 8C brings together these pieces of evidence into a hypothetical regulatory network. The network shows a two-step hierarchy, going from the ATM pathway, via CREB, to the nine most likely CREB target genes, which are deregulated between healthy and the damaged cells. Thus, we hypothesize that the ATM pathway indirectly deregulates those genes by deregulating CREB. In Additional file 2, Figure S7 B confronts the expression profile of the *CREB1 *gene with the expression profiles of its nine predicted targets.

## Discussion

In our approach, the information about the pathway topologies in the two analyzed cell populations is formalized in two simple models. Note that neither the pathway topologies nor the pathway models are intended to capture the dynamics and full spectrum of molecular interactions in signaling pathways. Instead, they are static and limited only to activatory signaling relations. Each model in a simplified way represents the knowledge of how the perturbations interrupt the flow of activations in the modeled pathway topology. The perturbations are required to turn the targeted regulator down (i.e., we do not model over-expressions). To relax these constraints the approach could be adapted to incorporate logical models (as applied by Szczurek *et al*. [44]), formalizing a broad range of signaling relations and allowing all possible perturbation experiments. Such extension would require distinguishing the experiments affecting a given regulator into two classes: one of experiments which down-regulate, and one of experiments which up-regulate the target genes of this regulator.

Unlike numerous approaches inferring gene regulation from expression data [45,46], here we do not measure the activity of the regulators from their expression levels. Concluding activity from expression has several drawbacks. First, TFs are often expressed on low mRNA levels and thus detection of their activity profile based on expression measurements may fail due to noise in the data. Second, regulator activity is modulated in many ways on post-translational level of signaling, by phosphorylation, ligand binding, degradation, etc. Thus, we follow Gat-Viks and Shamir [47] and Szczurek *et al*. [44] and derive the regulator activity from a given model of signaling pathway. The regulators are treated as proteins, and their activity in a given perturbation experiment depends on the perturbation and on signaling relations which exist on post-translational level. Thus, assuming the input pathway topologies are correct, the pathway models should encompass all means of influencing the regulators present in the two cell populations, such as phosphorylation or ligand binding.

This dependence on the pathway models implies that the correctness of the models is critical for the correctness of our results. JODA may fail when the input pathway topologies are insufficient. To assure high quality of the pathway models, they should first be confronted with available data and corrected using refinement procedures (see, for example, refinement strategy introduced by Gat-Viks and Shamir [47]). Moreover, the remaining genes (not the regulators) are measured from their expression levels, and their regulation is judged based on their transcriptional response to the perturbations of the regulators. The current view of regulation of gene expression in molecular biology [48] is more complex and includes, for example, post-transcriptional degradation by microRNAs. Ideally, our approach should integrate evidence of all means of gene regulation. We hope such integrative methods will be developed in the future. Importantly, our analysis can still be performed without any input knowledge. This option is valuable particularly in non-model organisms or under unusual experimental circumstances, where not much more is available other than newly generated expression data. In case when signaling relations between the regulators are not known, the input topologies given to JODA should be fully disconnected graphs. This corresponds to inferring regulator-target gene relations for each regulator independently, only based on the perturbation data for this regulator. In case when no regulator-target gene relations are given, JODA evaluates probabilities of differential expression (see the first step of the algorithm above) using unsupervised, instead of partially supervised mixture modeling [32]. However, as we show, incorporation of knowledge greatly improves the quality of deregulation analysis. Therefore, even if only partial information is available either about the signaling pathways, or about the target genes, it is still beneficial to provide it as input to JODA.

## Conclusions

Reprogramming of cellular character manifests itself in different cellular signaling, and, in a consequence, re-wiring of the downstream transcriptional network. JODA combines cell population-specific data and prior information from these interconnected levels. Moreover, deregulation is quantified in one score, merging effects from the two cell populations. Our results show advantage of JODA over investigating each cell population separately or without incorporation of prior knowledge.

JODA keeps the deregulation analysis in the strict biological context of pathway-induced gene regulation in the cell populations under study. To our knowledge, it is the first deregulation approach to take advantage of prior information about signaling pathway topology that differs between the compared populations of cells. The analyzed perturbations target common components of this signaling pathway in both cell populations. The known gene regulatory targets downstream of this pathway, specific for each cell population, are utilized as examples of differentially expressed genes.

In our analysis we focused on the deregulation between healthy cells and damaged cells treated with NCS. The obtained deregulation scores were further analyzed, first validating their congruence with the existing biological knowledge and next bringing new results. By finding functional clusters of the deregulated genes, we showed that the method assigns dominant deregulation scores to the genes playing important roles in the program of general response to DNA damage, in particular double strand breaks induced by NCS. Additionally, we investigated cooperativity between these deregulated genes, identifying known pathways and complexes in which the genes participate. We reviewed the DNA-damage related functionality of the genes with most extreme deregulation scores. Finally, we analyzed the indirect regulatory impact of the regulators in the ATM pathway on the genes in the functional clusters. An important advantage of our methodology is that it leads to testable mechanistic hypotheses. Here, we proposed a hierarchy of direct regulatory interactions by connecting the pathway to the deregulated DNA repair genes via the transcription factor CREB. Taken together, JODA is a step forward to a systems level, mechanistic understanding of changes in gene regulation between different cellular environments.

## Methods

### Perturbation dataset

We used the dataset of 30 expression measurements by Elkon *et al*. [28], in normal and in NCS-treated human HEK293 cells, composed of three replicates for each siRNA knockdown of ATM, RelA, and p53, and six replicates for control, in both cell populations (GEO series GSE1676, with 8794 genes measured). The raw data was normalized using quantile normalization and transformed into robust multi-array average expression values [49]. Quality of expression measurements was assessed with arrayQualityMetrics [50]. Four low-quality measurements were removed. We filtered out all genes without an ENTREZ identifier. In the case of multiple genes with the same identifier, we selected the one with the highest interquartile range (leaving 8498 genes). Consequent removal of outliers left 8463 genes. Next, we calculated vectors of log mean gene expression ratios for each knockdown versus control in both cell populations (averaging over repeats; together six vectors).

### Input TF-targets

Known target genes were collected for p53 both in the healthy and in the damaged cells, and for RelA only in the healthy cells (Additional file 1). For p53 in the damaged cells, we composed a set of 47 targets by selecting genes that have a *DNA repair *or *chromatin modification *function from experimentally verified p53 targets collected by Horvath *et al*. [51], the direct p53 targets detected with ChIP-PET and confirmed by expression analysis by Wei *et al*. [52], and finally by adding genes targeted by p53 upon ionizing radiation [53]. For p53 knockdown in the healthy cells, we took those verified targets of Horvath *et al*., and those direct p53 targets of Wei *et al*., which were not selected as targets in the damaged cells. Finally, for the analysis of RelA knockdown in the healthy cells we utilized a set of genes, identified using the ChIP-PET technology by Lim *et al*. [54], whose promoters are bound by RelA and contain an NF-*κ*B consensus motif.

### Differential expression probabilities

For each gene, we obtain a probability of differential expression upon each perturbation experiment using a belief-based partially supervised mixture modeling method implemented in an R package 'bgmm' [32]. For a given perturbation experiment, the method takes as input the data in a form of expression ratios and, if available, known examples of genes expected to be differentially expressed in this experiment. The examples are utilized to better fit a mixture of two Gaussians model to the expression data. In case when examples are not provided, unsupervised mixture modeling is applied instead. One Gaussian model component is interpreted as the cluster of differential genes, whereas the other as the cluster of genes which remained unchanged upon the perturbation as compared to control. Model-based clustering assigns each gene the probabilities to belong to the differential and to the unchanged cluster. The probability of differential expression for each gene is defined as the posterior probability to belong to the differential cluster. Here, the belief-based method is applied to each of the six vectors of log mean gene expression ratios for the knockdown experiments of RelA, ATM and p53 in the healthy and in the damaged cells (Additional file 2, Figure S8). For three of them the input TF-targets (see above) are used to define data points which are believed to belong to the differential cluster. The belief value is set to 0.95.

### Pathway topologies and models

The knowledge about a given pathway in a given cell population is first formalized in a graph, and next translated into a matrix showing how the perturbations affect the regulators. This formalism follows the idea of Nested Effects Models [55], where it is assumed that perturbation effects propagate through a given pathway. We denote the set of regulators as *V *= {*v*_1_, ..., *v_n_*}. The pathway topology in cell population *t *is a graph *G_t _*= (*V*, *A_t_*) with the set of nodes *V *and directed edges *A_t_*. There is an edge (*v_i_*, *v_j_*) ∈ *A_t _*whenever it is known that the pathway component *v_i _*activates *v_j _*in cell population *t*. *G_t _*may be cyclic and may have several connected components.

Internally, for each cell population separately, JODA utilizes the known pathway topologies to predict effects of perturbation experiments. Consider an experiment Δ*^t^v*, where a given regulator *v *is knocked down in a given cell population *t*. The regulator *v *together with all regulators, which are reachable from *v *in the pathway topology *t*, are called *affected *by the experiment Δ*^t^v*. The set of all experiments perturbing the regulators in *V *in cell population *t *is denoted *E_t_*. The predictions of affected regulators for all perturbations in *E_t _*are given by the transitive reflexive closure  of the pathway topology *G_t_*. To compute , we add an edge  whenever there exists a directed path from *v_i _*to *v_j _*in the pathway topology *G_t _*(including *v_i _*= *v_j_*, i.e., there are empty paths from each node to itself). The incidence matrix for  is called the *pathway model*, and is denoted . There is an entry 1 in row *v_i _*and column *v_j _*of the model matrix when , otherwise the entries are 0. In this way, an entry 1 tells that its row's perturbation affects its column's regulator. Thus, the set *E_v_*_,_*_t _*of all perturbation experiments that affect regulator *v *in cell population *t *is given by the rows of  which have an entry 1 in column *v*:(1)

This means that the set of affecting experiments *E_v_*_,_*_t _*contains both the perturbation of the regulator *v *itself, and perturbations of its upstream activators in the pathway. Assuming the model  is correct, the experiments in *E_v_*_,_*_t _*are expected to have a similar effect on the target genes of *v*.

### Regulation scores

To compute the regulation scores for a given regulator *v *∈ *V *and a given cell population *t*, the pathway model  is used.  defines the set *E_v_*_,_*_t _*(Eq.1) of experiments that affect the regulator *v *in *t*. The regulation scores (each lying in the [-1, 1] interval) are computed as an average over the signed probabilities of differential expression in the set of experiments *E_v_*_,_*_t_*:(2)

### Functional and pathway enrichment analysis

GO biological process categories as well as canonical pathways with fewer than 15 and more than 500 genes were excluded. Enrichment at the extremes of the ranked deregulation lists was computed using the GSEA algorithm [33] with default parameters. Only results with false discovery rate (FDR) ≤ 0.01 and family-wise error rate (FWER) ≤0.5 were considered significant.

### Functional clustering of GO terms and the resulting gene clustering

Similarity between the GO terms was assessed using the GOsim [56] implementation of the 'relevance' measure [34]. Next, the terms were hierarchically clustered by this similarity. We checked the possible clusterings with the number of clusters from five to twenty. For an assumed clustering size, the GO term clusters were formed by cutting the hierarchical clustering tree on a corresponding level. Next, from the functional clustering of GO terms, we obtained a functional clustering of genes, where each gene cluster corresponds to one GO term cluster. To this end, we collected the deregulated genes that are annotated with the terms from the GO term clusters, using the following procedure: First, for each GO term, we collected the corresponding deregulated genes in three steps: (i) Identify the deregulation lists in which this term is significantly overrepresented. (ii) From each identified deregulation list, collect the leading edge genes for this term, i.e., genes that contributed to the enrichment of the term in this list [33]. (iii) Take the intersection of the sets of genes collected from all lists identified for this term. Next, for each cluster, we took a union of the sets of genes collected for the terms in this cluster. The clustering size of both GO term and gene clusterings was set to thirteen. This number was chosen from a [5,20] interval such that the ratio of the number of clusters over the number of genes that are shared between the clusters is maximized (Additional file 2, Figure S3). Each of the resulting clusters was assigned a general name, summarizing the GO terms grouped in this cluster.

### Collection of genes from the enriched pathways

The deregulated genes, which belong to the enriched canonical pathways, were collected following the same three steps as for collection of genes for GO terms (steps (i)-(iii) above), but executed for each pathway.

### Complex enrichment analysis

Sets of genes forming each tested complex were downloaded from the Reactome database [57] (together 2816 complexes). For a set of genes in a given complex, and for a given deregulation list, we performed higher-tail hypergeometric enrichment tests iteratively for a number of 10 up to 500 most extreme (top or bottom) deregulated genes from the list. Finally, the minimum resulting *p*-value was selected to signify the enrichment of this complex in this deregulation list. Complexes with fewer than 15 genes, overlapping by less than 10 with the current set of deregulated genes in the iteration, were excluded. The size of the universe was set to all genes analyzed on the array (8498). Only results with the minimum enrichment *p*-value ≤ 10*^-^*^5 ^were considered significant.

### The *DNA repair *cluster network

66 genes in the *DNA repair *cluster are not included in canonical DNA damage response pathways. To characterize the connectivity between those genes, we applied SPIKE and Ingenuity. We identified together 126 relations connecting 52 of those genes either with each other or with other intermediate genes, complexes or protein families. SPIKE was applied to find all direct connections via at most a single intermediate node not included in the set. The connections may represent membership in a complex or regulation of different biochemical types, e.g. phosphorylation, protein-DNA (transcriptional) regulation, activation and protein-protein interaction. Ingenuity was applied to find interconnections restricting that each relationship was reported to appear for Human molecules, and that it is of one of the following types: expression, transcription, protein-DNA (all summarized as transcriptional regulation), activation, inhibition, membership, modification, phosphorylation, or protein-protein interaction. We collected all such direct relationships that are stored in the Ingenuity database. In addition, we applied Ingenuity to score known networks based on their enrichment in the input set of genes and collected all direct interactions present in the top three scoring networks (with scores 57, 45 and 18, respectively). The top scoring networks are related mostly to DNA replication, recombination, and repair, as well as tumor morphology, cell cycle and cell death. The networks have additional nodes that are not included in the input set but are highly connected to the genes in the set.

### TF binding prediction

For each functional cluster, we applied TransFind [42] to search the promoters of the genes in the cluster for over-representation of high-affinity binding of human TFs, which is conserved in their mouse orthologs. Given a set of genes, TransFind predicts TFs with affinities to the gene promoters significantly higher compared to a background set of genes (by default, all genes in the Ensembl57 database). Affinities are computed from a physical model, based on positional TF weight matrices. We tested a reduced set of human TRANSFAC [58] matrices, containing only a single, the most informative matrix for each TRANSFAC TF, setting all parameters to default. TransFind assesses the significance of binding to the promoters of a set of input genes with a multiple-testing-corrected (FDR) version of the Fisher's exact test. Only results with FDR ≤ 0.05 were considered significant.

## Competing interests

The authors declare that they have no competing interests.

## Authors' contributions

ES designed and implemented the algorithm, performed the analysis and wrote the paper. FM and IGV contributed ideas for the computational analysis, whereas PB contributed ideas for the method. FM assisted in writing the paper. JT and MV supervised the research. All authors read and approved the final manuscript.

## Supplementary Material

Additional file 1 **Known target genes given as input to JODA (listed in an Excel file)**. Click here for file

Additional file 2 **Figures S1-S8, Table S1**. Click here for file

Additional file 3 **Functional enrichment in the largest functional clusters (listed in an Excel file)**. Click here for file

Additional file 4 **Disease gene enrichment in the largest functional clusters (listed in an Excel file)**. Click here for file

Additional file 5 **Functional and disease enrichment in the genes most activated in DNA damage (listed in an Excel file)**. Click here for file

Additional file 6 **Interactions between the top most damage-activated genes (listed in an Excel file)**. Click here for file

## References

[B1] Chen X Cheung ST So S Fan ST Barry C Higgins J Lai KM Ji J Dudoit S Ng IOL Rijn MVD Botstein D Brown PO Gene expression patterns in human liver cancers Mol Biol Cell 2002 13 6 1929 39 10.1091/mbc.02-02-0023. 12058060 PMC117615

[B2] Golub TR Slonim DK Tamayo P Huard C Gaasenbeek M Mesirov JP Coller H Loh ML Downing JR Caligiuri MA Bloomfield CD Lander ES Molecular classification of cancer: class discovery and class prediction by gene expression monitoring Science 1999 286 5439 531 537 10.1126/science.286.5439.531 10521349

[B3] Nielsen TO West RB Linn SC Alter O Knowling MA O'Connell JX Zhu S Fero M Sherlock G Pollack JR Brown PO Botstein D van de Rijn M Molecular characterisation of soft tissue tumours: a gene expression study Lancet 2002 359 9314 1301 7 10.1016/S0140-6736(02)08270-3 11965276

[B4] Perou CM Sorlie T Eisen MB van de Rijn M Jeffrey SS Rees CA Pollack JR Ross DT Johnsen H Akslen LA Fluge O Pergamenschikov A Williams C Zhu SX Lonning PE Borresen-Dale AL Brown PO Botstein D Molecular portraits of human breast tumours Nature 2000 406 6797 747 52 10.1038/35021093 10963602

[B5] Sorlie T Tibshirani R Parker J Hastie T Marron JS Nobel A Deng S Johnsen H Pesich R Geisler S Demeter J Perou CM Lonning PE Brown PO Borresen-Dale AL Botstein D Repeated observation of breast tumor subtypes in independent gene expression data sets Proc Natl Acad Sci USA 2003 100 14 8418 23 10.1073/pnas.0932692100 12829800 PMC166244

[B6] Alizadeh AA Eisen MB Davis RE Ma C Lossos IS Rosenwald A Boldrick JC Sabet H Tran T Yu X Powell JI Yang L Marti GE Moore T Hudson J Lu L Lewis DB Tibshirani R Sherlock G Chan WC Greiner TC Weisenburger DD Armitage JO Warnke R Levy R Wilson W Grever MR Byrd JC Botstein D Brown PO Staudt LM Distinct types of diffuse large B-cell lymphoma identified by gene expression profiling Nature 2000 403 6769 503 511 10.1038/35000501 10676951

[B7] Garber ME Troyanskaya OG Schluens K Petersen S Thaesler Z Pacyna-Gengelbach M van de Rijn M Rosen GD Perou CM Whyte RI Altman RB Brown PO Botstein D Petersen I Diversity of gene expression in adenocarcinoma of the lung Proc Natl Acad Sci USA 2001 98 24 13784 9 10.1073/pnas.241500798 11707590 PMC61119

[B8] Minn AJ Gupta GP Siegel PM Bos PD Shu W Giri DD Viale A Olshen AB Gerald WL Massague J Genes that mediate breast cancer metastasis to lung Nature 2005 436 7050 518 24 10.1038/nature03799 16049480 PMC1283098

[B9] van de Vijver MJ He YD van't Veer LJ Dai H Hart AAM Voskuil DW Schreiber GJ Peterse JL Roberts C Marton MJ Parrish M Atsma D Witteveen A Glas A Delahaye L van der Velde T Bartelink H Rodenhuis S Rutgers ET Friend SH Bernards R A gene-expression signature as a predictor of survival in breast cancer N Engl J Med 2002 347 25 1999 2009 10.1056/NEJMoa021967 12490681

[B10] Wang SM Ooi LLPJ Hui KM Identification and validation of a novel gene signature associated with the recurrence of human hepatocellular carcinoma Clin Cancer Res 2007 13 21 6275 83 10.1158/1078-0432.CCR-06-2236 17975138

[B11] de la Fuente A From 'differential expression' to 'differential networking' - identification of dysfunctional regulatory networks in diseases Trends in genetics 2010 26 326 333 10.1016/j.tig.2010.05.001 20570387

[B12] Workman CT Mak HC McCuine S Tagne JB Agarwal M Ozier O Begley TJ Samson LD Ideker T A systems approach to mapping DNA damage response pathways Science 2006 312 5776 1054 1059 10.1126/science.1122088 16709784 PMC2811083

[B13] Mani KM Lefebvre C Wang K Lim WK Basso K Dalla-Favera R Califano A A systems biology approach to prediction of oncogenes and molecular perturbation targets in B-cell lymphomas Mol Syst Biol 2008 4 169 18277385 10.1038/msb.2008.2 PMC2267731

[B14] Taylor IW Linding R Warde-Farley D Liu Y Pesquita C Faria D Bull S Pawson T Morris Q Wrana JL Dynamic modularity in protein interaction networks predicts breast cancer outcome Nat Biotechnol 2009 27 2 199 204 10.1038/nbt.1522 19182785

[B15] Ergun A Lawrence CA Kohanski MA Brennan TA Collins JJ A network biology approach to prostate cancer Mol Syst Biol 2007 3 82 17299418 10.1038/msb4100125 PMC1828752

[B16] Schadt EE Molecular networks as sensors and drivers of common human diseases Nature 2009 461 7261 218 223 10.1038/nature08454 19741703

[B17] Hudson NJ Reverter A Dalrymple BP A differential wiring analysis of expression data correctly identifies the gene containing the causal mutation PLoS Comput Biol 2009 5 5 e1000382 10.1371/journal.pcbi.1000382 19412532 PMC2671163

[B18] Song L Kolar M Xing E KELLER: estimating time-varying interactions between genes Bioinformatics 2009 25 i128 10.1093/bioinformatics/btp192 19477978 PMC2687946

[B19] Barabasi AL Oltvai ZN Network biology: understanding the cell's functional organization Nat Rev Genet 2004 5 2 101 113 10.1038/nrg1272 14735121

[B20] Kostka D Spang R Finding disease specific alterations in the co-expression of genes Bioinformatics 2004 20 Suppl 1 i194 i199 10.1093/bioinformatics/bth909 15262799

[B21] Choi Y Kendziorski C Statistical methods for gene set co-expression analysis Bioinformatics 2009 25 10.1093/bioinformatics/btp502 PMC2781749 19689953

[B22] Tesson BM Breitling R Jansen RC DiffCoEx: a simple and sensitive method to find differentially coexpressed gene modules BMC Bioinformatics 2010 11 497 10.1186/1471-2105-11-497 20925918 PMC2976757

[B23] Gill R Datta S Datta S A statistical framework for differential network analysis from microarray data BMC Bioinformatics 2010 11 95 10.1186/1471-2105-11-95 20170493 PMC2838870

[B24] Ahmed A Xing EP Recovering time-varying networks of dependencies in social and biological studies Proceedings of the National Academy of Sciences of the United States of America 2009 106 10.1073/pnas.0901910106 PMC2704856 19570995

[B25] Ideker T Dutkowski J Hood L Boosting Signal-to-Noise in Complex Biology: Prior Knowledge Is Power Cell 2011 144 860 863 10.1016/j.cell.2011.03.007 21414478 PMC3102020

[B26] Ashburner M Ball CA Blake JA Botstein D Butler H Cherry JM Davis AP Dolinski K Dwight SS Eppig JT Harris MA Hill DP Issel-Tarver L Kasarskis A Lewis S Matese JC Richardson JE Ringwald M Rubin GM Sherlock G Gene ontology: tool for the unification of biology. The Gene Ontology Consortium Nat Genet 2000 25 25 9 10.1038/75556 10802651 PMC3037419

[B27] Gentleman RC Carey VJ Bates DM Bolstad B Dettling M Dudoit S Ellis B Gautier L Ge Y Gentry J Hornik K Hothorn T Huber W Iacus S Irizarry R Leisch F Li C Maechler M Rossini AJ Sawitzki G Smith C Smyth G Tierney L Yang JY Zhang J Bioconductor: open software development for computational biology and bioinformatics Genome Biol 2004 5 R80 10.1186/gb-2004-5-10-r80 15461798 PMC545600

[B28] Elkon R Rashi-Elkeles S Lerenthal Y Linhart C Tenne T Amariglio N Rechavi G Shamir R Shiloh Y Dissection of a DNA-damage-induced transcriptional network using a combination of microarrays, RNA interference and computational promoter analysis Genome Biol 2005 6 5 R43 10.1186/gb-2005-6-5-r43 15892871 PMC1175955

[B29] Povirk LF DNA damage and mutagenesis by radiomimetic DNA-cleaving agents: bleomycin, neocarzinostatin and other enediynes Mutat Res 1996 355 71 89 8781578 10.1016/0027-5107(96)00023-1

[B30] Kurz EU Lees-Miller SP DNA damage-induced activation of ATM and ATM-dependent signaling pathways DNA Repair (Amst) 2004 3 889 900 10.1016/j.dnarep.2004.03.029 15279774

[B31] Banin S Moyal L Shieh S Taya Y Anderson CW Chessa L Smorodinsky NI Prives C Reiss Y Shiloh Y Ziv Y Enhanced phosphorylation of p53 by ATM in response to DNA damage Science 1998 281 1674 1677 9733514 10.1126/science.281.5383.1674

[B32] Szczurek E Biecek P Tiuryn J Vingron M Introducing Knowledge into Differential Expression Analysis Journal of Computational Biology 2010 17 8 953 67 10.1089/cmb.2010.0034 20726790 PMC3122906

[B33] Subramanian A Tamayo P Mootha VK Mukherjee S Ebert BL Gillette MA Paulovich A Pomeroy SL Golub TR Lander ES Mesirov JP Gene set enrichment analysis: a knowledge-based approach for interpreting genome-wide expression profiles Proc Natl Acad Sci USA 2005 102 43 15545 15550 10.1073/pnas.0506580102 16199517 PMC1239896

[B34] Schlicker A Domingues FS Rahnenfuhrer J Lengauer T A new measure for functional similarity of gene products based on Gene Ontology BMC Bioinformatics 2006 7 302 10.1186/1471-2105-7-302 16776819 PMC1559652

[B35] Shiloh Y The ATM-mediated DNA-damage response: taking shape Trends Biochem Sci 2006 31 7 402 10 10.1016/j.tibs.2006.05.004 16774833

[B36] Lavin MF Kozlov S ATM activation and DNA damage response Cell Cycle 2007 6 8 931 42 10.4161/cc.6.8.4180 17457059

[B37] Hoeijmakers JHJ DNA damage, aging, and cancer N Engl J Med 2009 361 15 1475 85 10.1056/NEJMra0804615 19812404

[B38] Elkon R Vesterman R Amit N Ulitsky I Zohar I Weisz M Mass G Orlev N Sternberg G Blekhman R Assa J Shiloh Y Shamir R SPIKE-a database, visualization and analysis tool of cellular signaling pathways BMC Bioinformatics 2008 9 110 10.1186/1471-2105-9-110 18289391 PMC2263022

[B39] Wood RD Mitchell M Lindahl T Human DNA repair genes, 2005 Mutat Res 2005 577 1-2 275 283 15922366 10.1016/j.mrfmmm.2005.03.007

[B40] Honma M Sakuraba M Koizumi T Takashima Y Sakamoto H Hayashi M Non-homologous end-joining for repairing I-SceI-induced DNA double strand breaks in human cells DNA Repair (Amst) 2007 6 781 788 10.1016/j.dnarep.2007.01.004 17296333

[B41] Seluanov A Mao Z Gorbunova V Analysis of DNA double-strand break (DSB) repair in mammalian cells J Vis Exp 2010 8 43 10.3791/2002 PMC3157866 20864925

[B42] Kielbasa S Klein H Roider H Vingron M Blüthgen N TransFind-predicting transcriptional regulators for gene sets Nucleic Acids Research 2010 38 Web Server W275 W280 10.1093/nar/gkq438 PMC2896106 20511592

[B43] Zhang X Odom DT Koo SH Conkright MD Canettieri G Best J Chen H Jenner R Herbolsheimer E Jacobsen E Kadam S Ecker JR Emerson B Hogenesch JB Unterman T Young RA Montminy M Genome-wide analysis of cAMP-response element binding protein occupancy, phosphorylation, and target gene activation in human tissues Proc Natl Acad Sci USA 2005 102 12 4459 4464 10.1073/pnas.0501076102 15753290 PMC555478

[B44] Szczurek E Gat-Viks I Tiuryn J Vingron M Elucidating regulatory mechanisms downstream of a signaling pathway using informative experiments Molecular Systems Biology 2009 5 10.1038/msb.2009.45 PMC2724975 19584836

[B45] Markowetz F Spang R Inferring cellular networks-a review BMC Bioinformatics 2007 8 Suppl 6 S5 10.1186/1471-2105-8-S6-S5 17903286 PMC1995541

[B46] Markowetz F How to understand the cell by breaking it: network analysis of gene perturbation screens PLoS Comput Biol 2010 6 2 e1000655 10.1371/journal.pcbi.1000655 20195495 PMC2829042

[B47] Gat-Viks I Shamir R Refinement and expansion of signaling pathways: the osmotic response network in yeast Genome Res 2007 17 3 358 367 10.1101/gr.5750507 17267811 PMC1800927

[B48] Shafer G Ed Post-transcriptional gene regulation 2008 Humana press

[B49] Irizarry RA Bolstad BM Collin F Cope LM Hobbs B Speed TP Summaries of Affymetrix GeneChip probe level data Nucleic Acids Res 2003 31 4 e15 10.1093/nar/gng015 12582260 PMC150247

[B50] Kauffmann A Gentleman R Huber W arrayQualityMetrics-a bioconductor package for quality assessment of microarray data Bioinformatics 2009 25 3 415 6 10.1093/bioinformatics/btn647 19106121 PMC2639074

[B51] Horvath MM Wang X Resnick MA Bell DA Divergent evolution of human p53 binding sites: cell cycle versus apoptosis PLoS Genet 2007 3 7 e127 10.1371/journal.pgen.0030127 17677004 PMC1934401

[B52] Wei CL Wu Q Vega VB Chiu KP Ng P Zhang T Shahab A Yong HC Fu Y Weng Z Liu J Zhao XD Chew JL Lee YL Kuznetsov VA Sung WK Miller LD Lim B Liu ET Yu Q Ng HH Ruan Y A global map of p53 transcription-factor binding sites in the human genome Cell 2006 124 207 19 10.1016/j.cell.2005.10.043 16413492

[B53] Jen KY Cheung VG Identification of novel p53 target genes in ionizing radiation response Cancer Res 2005 65 17 7666 73 16140933 10.1158/0008-5472.CAN-05-1039

[B54] Lim CA Yao F Wong JJY George J Xu H Chiu KP Sung WK Lipovich L Vega VB Chen J Shahab A Zhao XD Hibberd M Wei CL Lim B Ng HH Ruan Y Chin KC Genome-wide mapping of RELA(p65) binding identifies E2F1 as a transcriptional activator recruited by NF-kappaB upon TLR4 activation Mol Cell 2007 27 4 622 35 10.1016/j.molcel.2007.06.038 17707233

[B55] Markowetz F Bloch J Spang R Non-transcriptional pathway features reconstructed from secondary effects of RNA interference Bioinformatics 2005 21 21 4026 4032 10.1093/bioinformatics/bti662 16159925

[B56] Frohlich H Speer N Poustka A Beissbarth T GOSim-an R-package for computation of information theoretic GO similarities between terms and gene products BMC Bioinformatics 2007 8 166 10.1186/1471-2105-8-166 17519018 PMC1892785

[B57] Vastrik I D'Eustachio P Schmidt E Joshi-Tope G Gopinath G Croft D de Bono B Gillespie M Jassal B Lewis S Matthews L Wu G Birney E Stein L Reactome: a knowledge base of biologic pathways and processes Genome Biol 2007 8 3 R39 10.1186/gb-2007-8-3-r39 17367534 PMC1868929

[B58] Wingender E Dietze P Karas H Knüppel R TRANSFAC: a database on transcription factors and their DNA binding sites Nucleic Acids Res 1996 24 238 241 10.1093/nar/24.1.238 8594589 PMC145586

