# Supplementary material for: Deregulation upon DNA damage revealed by joint analysis of context-specific perturbation data
Source: BMC Bioinformatics. 2011 Jun 21;12:249. doi: 10.1186/1471-2105-12-249 (PMC3236061; doi:10.1186/1471-2105-12-249)
Supplement: Additional file 2 — Figures S1-S8, Table S1. [file 1471-2105-12-249-S2.PDF]

Deregulation upon DNA damage revealed by joint analysis of context-specific perturbation data

Ewa Szczurek[1,2,3,*], Florian Markowetz[4], Irit Gat-Viks[5], Przemysław Biecek[3], Jerzy Tiuryn[3] and Martin Vingron[1]

[1]Computational Molecular Biology Department, Max Planck Institute for Molecular Genetics, Ihnestrasse 73, 14195 Berlin, Germany,

[2]International Max Planck Research School for Computational Biology and Scientific Computing, Berlin, Germany,

[3]Faculty of Mathematics, Informatics and Mechanics, University of Warsaw, Banacha 2, 02-097 Warsaw, Poland.

[4]Cancer Research UK Cambridge Research Institute, Cambridge, United Kingdom,

[5]Broad Institute of MIT and Harvard, 7 Cambridge Center, Cambridge, MA 02142, USA.

[*]Corresponding author. E-mail: szczurek@molgen.mpg.de

Additional file 2

List of items:

Figure S1 Distribution of deregulation scores....................2

Figure S2 Functional gene cluster sizes and overlaps....................3

Figure S3 Choice of the number of clusters....................3

Figure S4 Functional clusters versus the network of NCS action....................4

Figure S5 Deregulation versus regulation list enrichment analysis....................5-7

Figure S6 Deregulation p-values for the most activated genes....................8

Figure S7 Expression profiles....................9

Figure S8 Partially supervised gaussian mixture modeling with bgmm....................10

Table S1 Abbreviations of complex names....................11

REFERENCES....................11

Figure S1

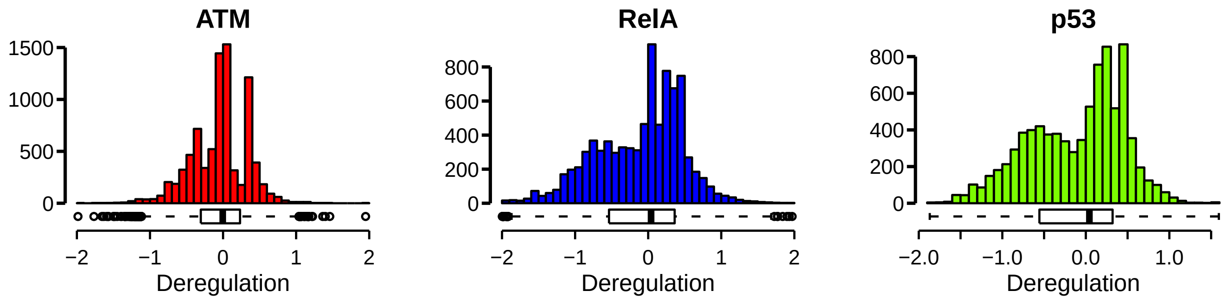

Fig. 1: Distribution of the deregulation scores. Histograms of the deregulation scores of all measured genes for the regulators ATM, RelA and p53. Below each histogram, a boxplot of the scores is shown.

Figure S2

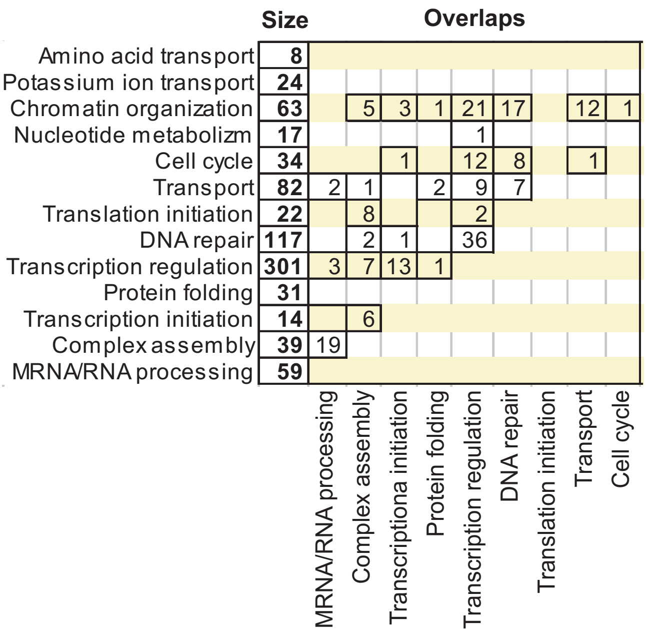

Fig. 2: Functional gene cluster sizes and overlaps. The matrix represents the clusters (rows), their sizes (Size column), and their pairwise overlaps (Overlaps matrix). Columns of the overlaps matrix correspond only to those clusters that do overlap with any other cluster. Entries show the non-zero number of genes in the overlap and otherwise are empty.

Figure S3

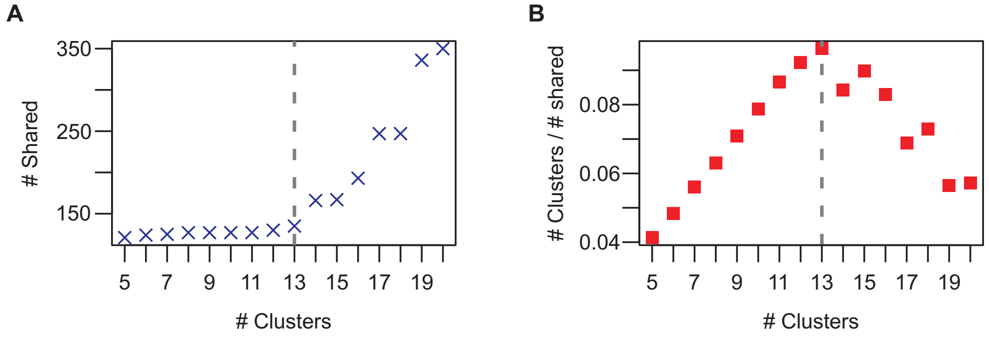

Fig. 3: Choice of the number of clusters. (**A**) The total number of unique genes shared between the clusters, traced over all clusterings with sizes from five to twenty. The set of genes shared between the clusters is obtained as the union of all pairwise cluster intersections. The number of shared genes increases with the size of the clustering. (**B**) The number of clusters in our functional clustering is selected from the $[5; 20]$ interval so that the ratio of the number of clusters over the number of shared genes is maximized. The more clusters, the more functions are represented in the clustering (one cluster groups a set of GO terms). The less shared genes, the smaller overall overlap between the clusters. Gray dashed line in **A**, **B** marks the selected cluster number (thirteen).

Figure S4

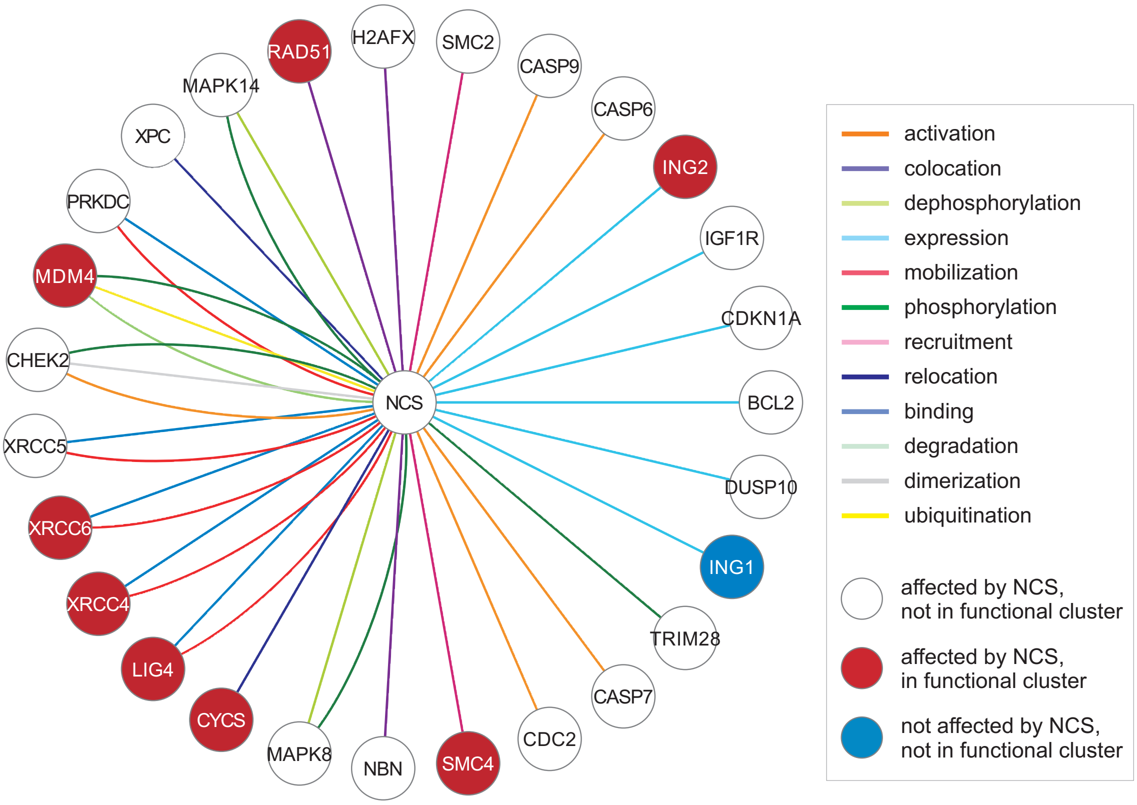

Fig. 4: Functional clusters versus the network of NCS action. The graph connects NCS (neocarzinostatin; center node) to its gene targets. Edges are colored by the type of NCS action on the genes. Nodes corresponding to the affected genes are colored by their agreement with functional clusters. Genes colored red are affected by NCS and are present in the functional clusters. Gene colored blue is known not to be affected and is not contained in the clusters. Genes colored white are affected by NCS and are not contained in the functional clusters. In majority of cases, the presence or absence of genes in the functional clusters agrees with what is known about NCS acting and what is known about NCS *not* acting on the genes. For example, NCS increases expression of *ING2* [5], and it is deregulated in our system. On the other hand, *ING1* is not deregulated and is expected not to, as it was reported to show very little or no change in expression upon NCS treatment [5]. In disagreement with the current knowledge, the functional clusters do not contain *PRKDC*. Still, they do contain genes coding for members of the protein-protein complex consisting of the DNA ligase 4 (LIG4) and XRCC4, that is mobilized by NCS in a PRKDC-dependent manner [2]. Surprisingly, from the two proteins (SMC2 and SMC4) which upon NCS treatment are recruited to chromatin from Hela cells, only SMC4 is coded by a gene which belongs to the functional clusters [1]. The deregulated functional clusters do not contain two apoptosis genes, *BCL2* and *CDKN1A*, which were reported to change expression under NCS treatment in MCF7 cells [3]. As we show in Figure 4 A, apoptosis pathway is overrepresented in the deregulated genes, but mostly the part which is also functional in DNA repair and transcription regulation. Interestingly, the deregulated clusters contain also *MDM4* and *RAD51*. NCS increases phosphorylation of MDM4 protein, that is dependent on ATM [6]. In nuclear foci, NCS promotes co-localization of RAD51 protein and phosphorylated p53 [4]. Deregulation of *MDM4* and *RAD51* means that not only their protein products change upon NCS treatment, but also they switch between untreated and treated cells on transcriptional level. Other proteins known to be phosphorylated upon NCS treatment (CHEK2, TRIM28, MAPK8, MAPK14) are not coded by deregulated genes. Similarly, none of the genes coding for proteins known to be activated by NCS are deregulated.

Figure S5

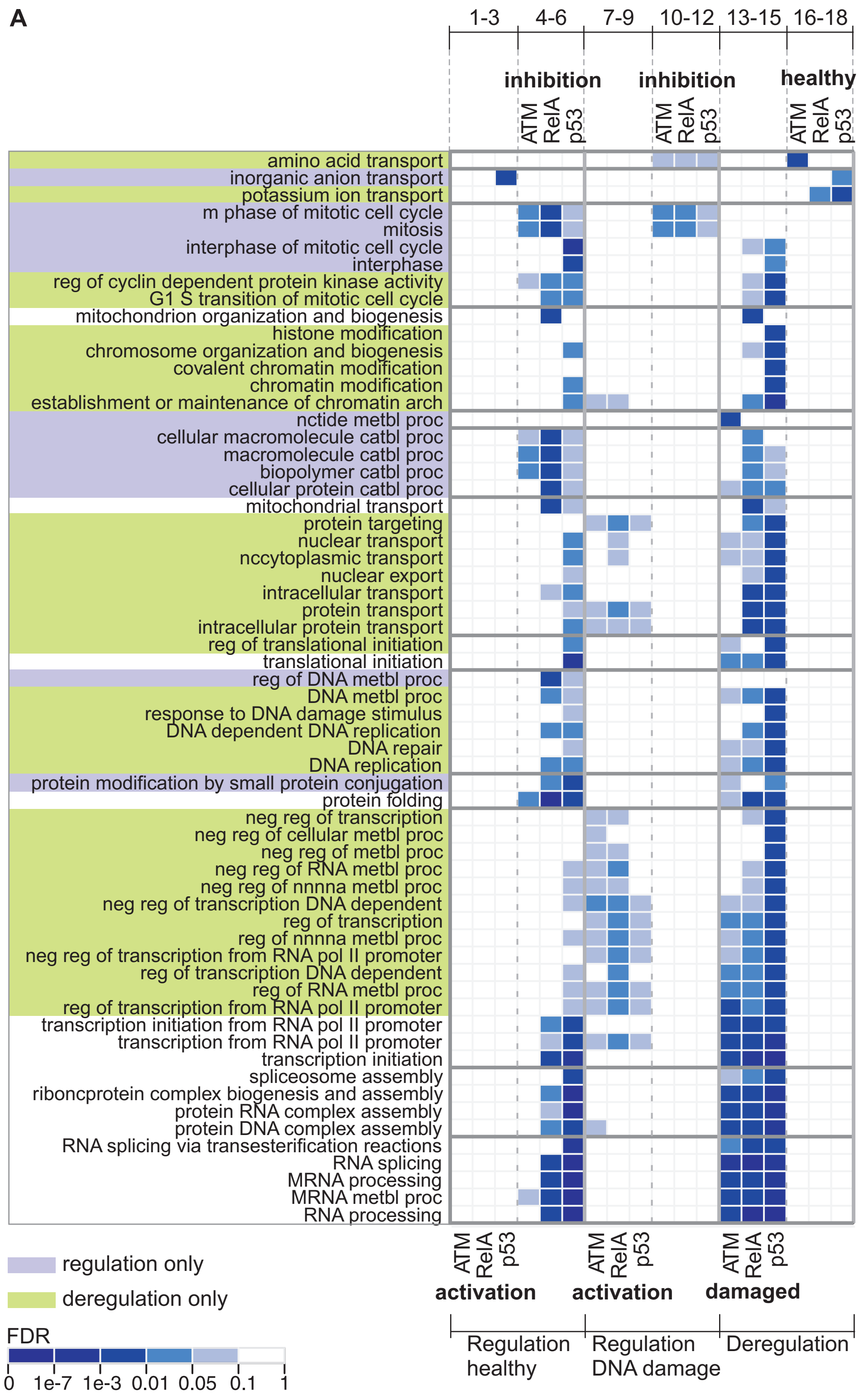
A
1-3
4-6
7-9
10-12
13-15
16-18
inhibition
inhibition
healthy
ATM
RelA
p53
amino acid transport
inorganic anion transport
potassium ion transport
m phase of mitotic cell cycle
mitosis
interphase of mitotic cell cycle
interphase
reg of cyclin dependent protein kinase activity
G1 S transition of mitotic cell cycle
mitochondrion organization and biogenesis
histone modification
chromosome organization and biogenesis
covalent chromatin modification
chromatin modification
establishment or maintenance of chromatin arch
nctide metbl proc
cellular macromolecule catbl proc
macromolecule catbl proc
biopolymer catbl proc
cellular protein catbl proc
mitochondrial transport
protein targeting
nuclear transport
nccytoplasmic transport
nuclear export
intracellular transport
protein transport
intracellular protein transport
reg of translational initiation
translational initiation
reg of DNA metbl proc
DNA metbl proc
response to DNA damage stimulus
DNA dependent DNA replication
DNA repair
DNA replication
protein modification by small protein conjugation
protein folding
neg reg of transcription
neg reg of cellular metbl proc
neg reg of metbl proc
neg reg of RNA metbl proc
neg reg of nnnna metbl proc
neg reg of transcription DNA dependent
reg of transcription
reg of nnnna metbl proc
neg reg of transcription from RNA pol II promoter
reg of transcription DNA dependent
reg of RNA metbl proc
reg of transcription from RNA pol II promoter
transcription initiation from RNA pol II promoter
transcription from RNA pol II promoter
transcription initiation
spliceosome assembly
riboncprotein complex biogenesis and assembly
protein RNA complex assembly
protein DNA complex assembly
RNA splicing via transesterification reactions
RNA splicing
MRNA processing
MRNA metbl proc
RNA processing
regulation only
deregulation only
FDR
0
1e-7
1e-3
0.01
0.05
0.1
1
activation
activation
damaged
Regulation healthy
Regulation DNA damage
Deregulation

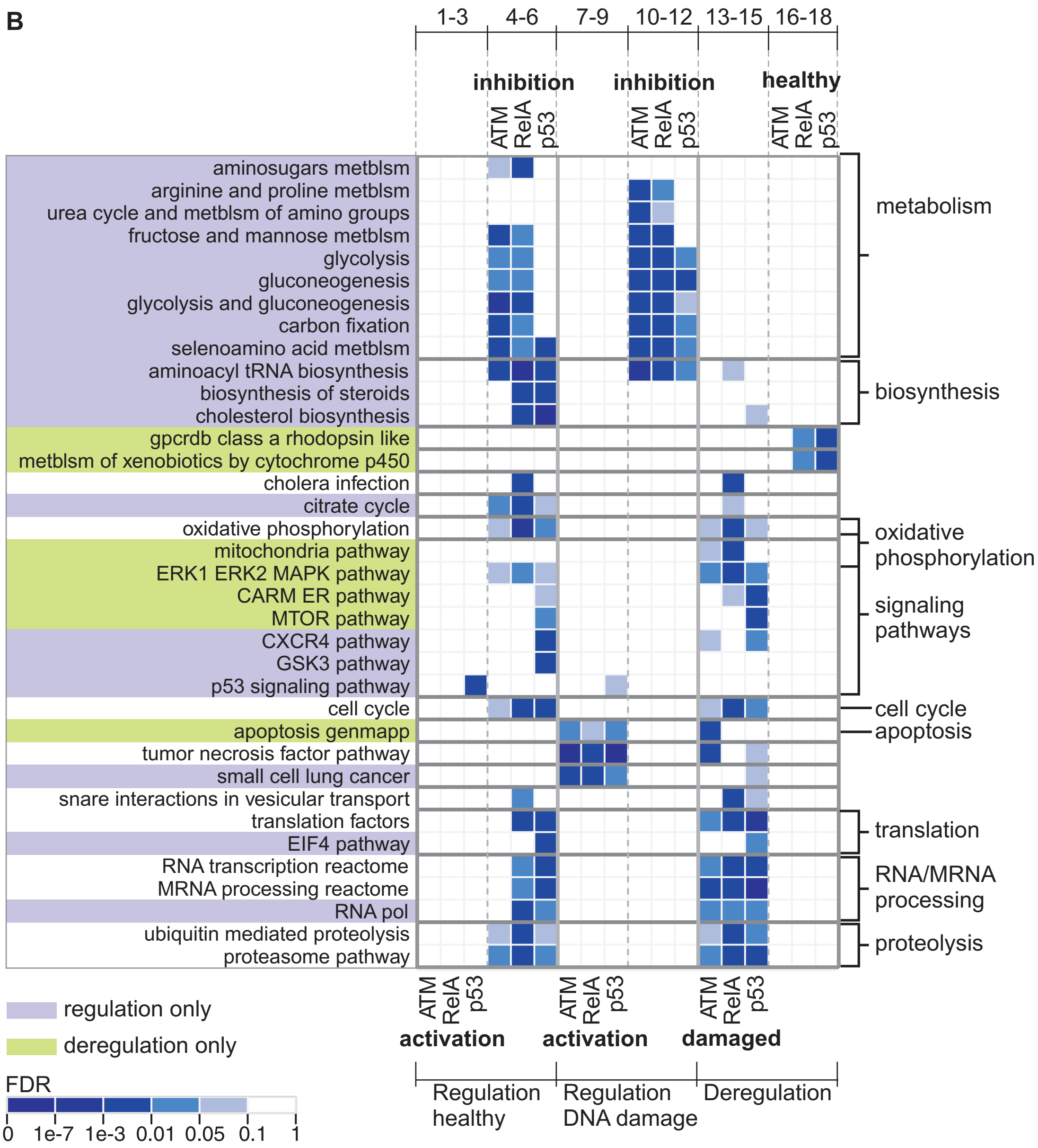
B
1-3
4-6
7-9
10-12
13-15
16-18
inhibition
inhibition
healthy
ATM
RelA
p53
aminosugars metblsm
arginine and proline metblsm
urea cycle and metblsm of amino groups
fructose and mannose metblsm
glycolysis
gluconeogenesis
glycolysis and gluconeogenesis
carbon fixation
selenoamino acid metblsm
aminoacyl tRNA biosynthesis
biosynthesis of steroids
cholesterol biosynthesis
gpcrdb class a rhodopsin like
metblsm of xenobiotics by cytochrome p450
cholera infection
citrate cycle
oxidative phosphorylation
mitochondria pathway
ERK1 ERK2 MAPK pathway
CARM ER pathway
MTOR pathway
CXCR4 pathway
GSK3 pathway
p53 signaling pathway
cell cycle
apoptosis genmapp
tumor necrosis factor pathway
small cell lung cancer
snare interactions in vesicular transport
translation factors
EIF4 pathway
RNA transcription reactome
MRNA processing reactome
RNA pol
ubiquitin mediated proteolysis
proteasome pathway
metabolism
biosynthesis
oxidative phosphorylation
signaling pathways
cell cycle
apoptosis
translation
RNA/MRNA processing
proteolysis
activation
activation
damaged
Regulation healthy
Regulation DNA damage
Deregulation
regulation only
deregulation only
FDR
0
1e-7
1e-3
0.01
0.05
0.1
1

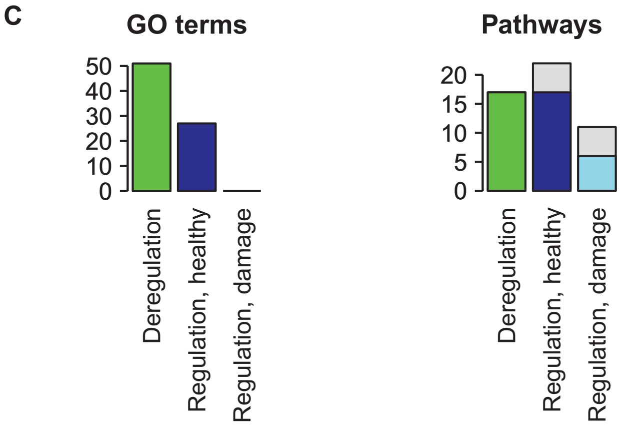

Fig. 5: The joint versus separate approach. A comparison of the functional (GO term) and pathway enrichment analysis of deregulation scores to an alternative approach, which is to study the enrichment in the lists of sorted regulation scores independently. A sorted list of regulation scores contains on the one end the genes which are up-regulated by the regulator perturbation (indirectly inhibited by the regulator) and on the other the genes that are down-regulated by the perturbation (indirectly activated by the regulator). (**A**) Functional enrichment. The rows of the matrix correspond to the enriched GO terms. The columns correspond to the extremes (top or bottoms) of the gene lists sorted by the regulation scores (shortly, regulation lists) for the healthy cells (first six columns), the extremes of the regulation lists for the damaged cells (middle six columns) and the extremes of the deregulation lists (last six columns). Columns one to three: genes that are activated (have very low regulation scores) by ATM, RelA and p53 in the healthy cells. Four to six: genes that are inhibited (have very high regulation scores) in the healthy cells. Seven to nine: genes that are activated in the damaged cells. Ten to twelve: genes that are inhibited in the damaged cells. Thirteen to fifteen: genes that are more activated in the damaged cells. Sixteen to eighteen: genes more activated in the healthy cells. Each GO term shown is significantly enriched in at least one column with FDR$\leq$ 0.01 and FWER$\leq$ 0.5. FDR (entries of the matrix; identified using GSEA) is indicated in shades of blue. The enriched terms were grouped into thirteen clusters by similarity (Methods), and sorted by the average enrichment in the columns thirteen to fifteen. Terms significantly enriched only in any extreme of the regulation lists, and not in any extreme of the deregulation lists, are marked with violet background. Terms significantly enriched only in any extreme of the deregulation lists, and not in any extreme of the regulation lists, are marked with green background. Terms significantly enriched both in any extreme of the regulation lists, and in any extreme of the deregulation lists, are marked with white background. mtbl, metabolic; nc, nucleo; pol, polymerase; reg, regulation; neg, negative; pos, positive; proc, process; arch, architecture; nnnna, nucleobase, nucleoside, nucleotide, and nucleic acid. (**B**) Pathway enrichment. The matrix as in **A**, only the rows correspond to the enriched canonical pathways (identified using GSEA). (**C**) Boxplots summarizing the number of enriched GO terms (left) and pathways (right). The enrichment in any of the deregulation lists (green) is compared to the enrichment in any of the regulation lists in the healthy cells (dark blue and gray) as well as any of the regulation lists in the damaged cells (light blue and gray). For the regulation lists, the gray fraction of the bars indicates the number of those GO terms or pathways, which are not interesting for the shift between the healthy and the damaged cells. Such terms or pathways are enriched in genes which show the same effect to the perturbations (i.e., are regulated in the same way) in the heatlhy and in the damaged cells. One example is the glycolysis pathway (compare in **B**), enriched in the genes that are inhibited both in the healthy cells and in the damaged cells.

Figure S6

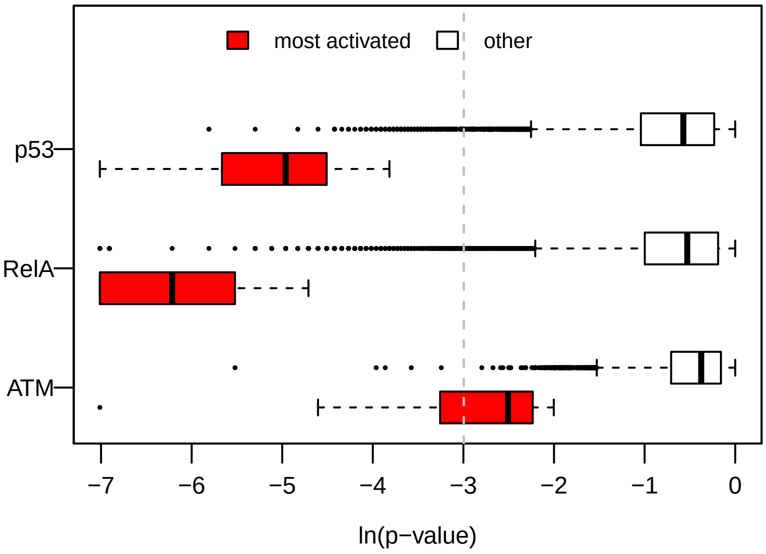

Fig. 6: Deregulation p-values for the most activated genes. The boxplots show distributions of ln(p-values) calculated for deregulation scores. For each regulator, its top one hundred most activated genes are denoted as 'most activated' and their p-values are summarized by red boxplots. P-values for all other genes are represented by white boxplots. The dashed vertical line denotes significance threshold 0.05. For the genes most activated by RelA and p53 the statistical analysis confirms that deregulation scores are significantly different than zero. For ATM the deregulation scores of the top most activated genes are less significant. As expected, the outliers of the distributions of other genes suggest that apart from the top one-hundred there are more significantly deregulated genes. In particular, it is expected that the genes most activated in the healthy cells (with deregulation scores on the opposite extreme of the deregulation lists) have significant scores. Still, for the three regulators the p-values of almost all other genes are not significant, which assures that there are no artifacts caused by the testing procedure.

Figure S7

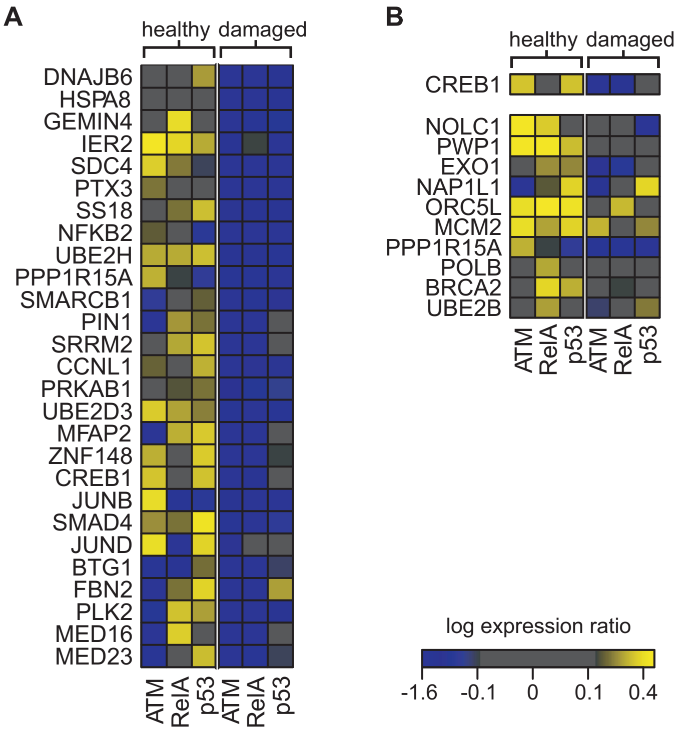

Fig. 7: Expression profiles. Matrices show expression profiles of genes (rows) across the six analyzed perturbation experiments (from left to right, knockdowns of ATM, RelA and p53 in the healthy and in the damaged cells; columns). (**A**) Expression profiles of those 28 genes most activated in DNA damage which are presented in Figure 6. As expected, majority of the genes is up-regulated by the knockdowns in the healthy, and down-regulated in the damaged cells (i.e., is inhibited by the regulators in the healthy cells and activated in the damaged cells). (**B**) An expression profile for CREB (top) and expression profiles of its nine likely target genes (see Figure 8). The regulatory connection between CREB1 and the proposed target genes would not be concluded from the correlation of their expression profiles. However, in some cases, visual inspection of the expression data suggests decorrelation (i.e., gain or loss of correlation between the cell populations). For example, expression profile of *EXO1* is not strongly correlated with expression of *CREB1* in the healthy cells, but in the damaged cells their profiles are identical. Similarly, there is a gain of correlation in the case of *PPP1R15A*. Still, for most of the targets there is no obvious connection between the expression profile of *CREB1* and expression of the likely targets. Thus, the proposed deregulation of CREB transcription factor activity and consequent deregulation of genes must include a higher level of control, and not all means of target gene activation or deactivation can be explained by expression data alone.

Figure S8

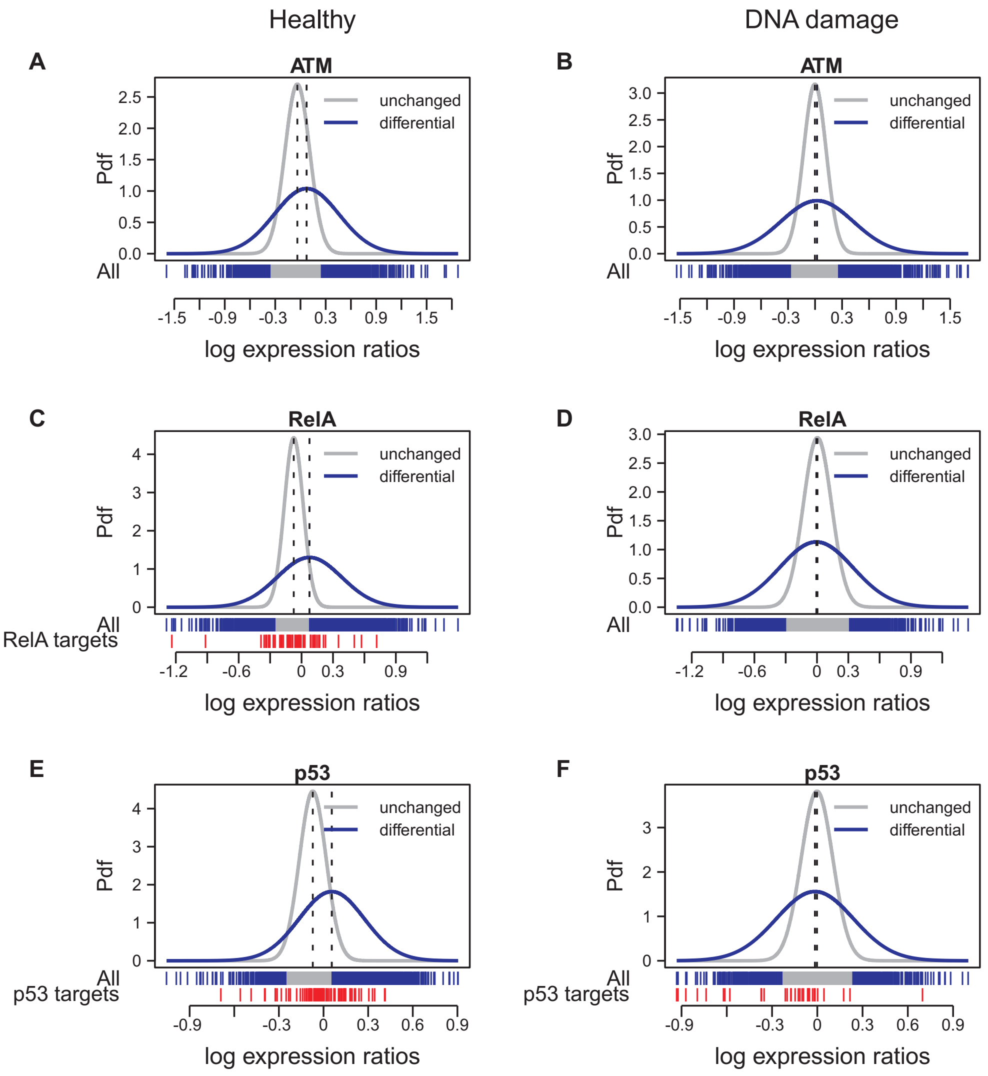

Fig. 8: Partially supervised gaussian mixture modeling with the R package 'bgmm', applied to the perturbations of ATM, RelA and p53 in the healthy and in the damaged cells. (**A**) **Left.** Two gaussian components (grey for the unchanged genes and blue for the differential genes) of the mixture model fitted to the data (log expression ratios, ΔATM over control in the healthy cells). Below: data points colored according to the corresponding model based clustering. Genes can be clustered as differential or unchanged based on their most probable gaussian component. **Right:** Plot as on the left, but the data are for the damaged cells. (**B**) Plots as in **A** but for the knockdowns of RelA in the healthy and damaged cells. (**C**) Plots as in **A** but for the knockdowns of p53. In **B** left and **C** the data points marked in red below the plots indicate the input known target genes, which are expected to be differentially expressed upon their transcription factor knockdowns.

Table S1

Complex	Abbreviation
EJ	Exon junction
SA	Spliceosomal A
SB	Spliceosomal B
SC	Spliceosomal Active C/ Spliceosomal Intermediate C/
	Spliceosomal active C with lariat containing 5-end cleaved pre-mRNP:CBC
ATAC B/C	ATAC B/ ATAC C/ ATAC C with lariat containing 5-end cleaved mRNA

Table 1. Abbreviations of complex names. "/" lists the overlapping complexes that have identical enrichment p-values and which are abbreviated with the same name.

REFERENCES

[1]M. Blank, Y. Lerenthal, L. Mittelman, and Y. Shiloh. Condensin I recruitment and uneven chromatin condensation precede mitotic cell death in response to DNA damage. *J. Cell Biol.*, 174:195–206, Jul 2006.

[2]J. Drouet, C. Delteil, J. Lefrancois, P. Concannon, B. Salles, and P. Calsou. DNA-dependent protein kinase and XRCC4-DNA ligase IV mobilization in the cell in response to DNA double strand breaks. *J. Biol. Chem.*, 280:7060–7069, Feb 2005.

[3]Y. Liang, C. Yan, and N. F. Schor. Apoptosis in the absence of caspase 3. *Oncogene*, 20:6570–6578, Oct 2001.

[4]S. P. Linke, S. Sengupta, N. Khabie, B. A. Jeffries, S. Buchhop, S. Miska, W. Henning, R. Pedeux, X. W. Wang, L. J. Hofseth, Q. Yang, S. H. Garfield, H. W. Sturzbecher, and C. C. Harris. p53 interacts with hRAD51 and hRAD54, and directly modulates homologous recombination. *Cancer Res.*, 63:2596–2605, May 2003.

[5]M. Nagashima, M. Shiseki, K. Miura, K. Hagiwara, S. P. Linke, R. Pedeux, X. W. Wang, J. Yokota, K. Riabowol, and C. C. Harris. DNA damage-inducible gene p33ING2 negatively regulates cell proliferation through acetylation of p53. *Proc. Natl. Acad. Sci. U.S.A.*, 98:9671–9676, Aug 2001.

[6]Y. Pereg, D. Shkedy, P. de Graaf, E. Meulmeester, M. Edelson-Averbukh, M. Salek, S. Biton, A. F. Teunisse, W. D. Lehmann, A. G. Jochemsen, and Y. Shiloh. Phosphorylation of Hdmx mediates its Hdm2- and ATM-dependent degradation in response to DNA damage. *Proc. Natl. Acad. Sci. U.S.A.*, 102:5056–5061, Apr 2005.
